# Tailoring K^+^ Dosage in K^+^/Zn^2+^ Mixed Electrolytes via Lattice Expansion Regulation of Zinc Hexacyanoferrate for High‐performance Zinc Ion Batteries

**DOI:** 10.1002/advs.75876

**Published:** 2026-06-24

**Authors:** Yewei Li, Yuqian Li, Yuchen Zhang, Shuang Zheng, Yanhao Pan, Jiyuan You, Yan Liu, Wenju Wang

**Affiliations:** ^1^ School of Energy and Power Engineering Nanjing University of Science and Technology Nanjing China; ^2^ School of Chemistry and Chemical Engineering Nanjing University of Science and Technology Nanjing China; ^3^ Huaneng Nanjing Jinling Power Generation Co., Ltd. Nanjing China

**Keywords:** aqueous zinc ion batteries, aqueous Zn based electrolyte, cation electrolyte additive, ion competition, PBA cathode

## Abstract

Incorporating K^+^ into Zn‐based electrolytes can enhance the performance of aqueous zinc ion batteries, while the lack of a clear rule for matching specific K^+^ dosage with the cathode lattice regulation hinders its practical advancement. Herein, we propose a current‐dependent critical dissolution concentration (CDC) as a quantitative reference to optimize K^+^ content, aiming to realize suppressed cyclic degradation of zinc hexacyanoferrate (ZnHCF) cathodes through precise lattice expansion control. The spontaneous dissolution of ZnHCF is theoretically analyzed via *Van't Hoff isothermal equation*, and a quantitative relationship between ZnHCF dissolution and K^+^ concentration is obtained. Further, HRTEM and XRD confirm that the K^+^ concentration below the current‐free CDC causes ZnHCF dissolution due to the current enrichment effect, indicating that K^+^ concentration above CDC can trigger spontaneous ZnHCF dissolution, while K^+^ concentration is lower than CDC, the ZnHCF layer distance is not sufficiently expanded. Based on this, the long‐term cycling performance of gradient K^+^ concentration under gradient current density and in situ electrode stability tests quantifies the optimal K^+^ ratio at each current density and successfully predicts the optimal K^+^ concentration corresponding to a certain current density. Overall, this work advances general design guidelines for alkali metal cation additives in aqueous zinc ion batteries.

## Graphical abstract

The study proposes a fitting curve as a reference to quantify the optimal K^+^ ratio in K^+^/Zn^2+^ mixed electrolyte, thus achieving stable and high‐capacity aqueous zinc ion batteries at each current density.

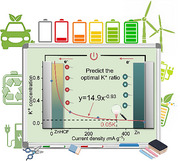

## 1 Introduction

In the post‐lithium energy field, safe and efficient energy storage systems have become an urgent demand due to the rapid development of renewable energy [1, 2, 3, 4] and electric transportation [5, 6, 7, 8]. Aqueous zinc ion batteries (AZIBs) have become new candidates for future energy storage technologies due to their low cost, environmental friendliness, and high theoretical capacity [9, 10, 11, 12, 13, 14]. However, the electrochemical stability of AZIBs still faces significant challenges, especially the cathode stability in aqueous electrolytes [15, 16, 17, 18, 19]. Typically, zinc hexacyanoferrate (ZnHCF), belonging to Prussian blue analogs (PBAs), has broad application in AZIBs due to high voltage and adjustable framework [20, 21, 22, 23, 24, 25], while low specific capacity and rapid capacity fading in conventional dilute aqueous electrolytes seriously limit its practical application, which has been widely verified in previous representative works [26, 27, 28, 29, 30, 31].

In previous studies, the introduction of alkali metal cations (Li^+^, Na^+^ and K^+^) into the electrolyte has been shown as an effectively strategy in improving the performance of AZIBs: (1) K^+^ preferentially inserts/extracts from ZnHCF cathode channel in both thermodynamics and kinetics compared with Zn^2+^, resulting in excellent rate performance in K‐Zn hybrid ion batteries [32, 33, 34]; (2) K^+^ has higher ion mobility and self‐diffusion coefficient compared with Zn^2+^, which can accelerate ion migration and optimize the electric double layer (EDL) [35, 36, 37, 38]; (3) K^+^ acts as an electrostatic shield on the Zn anode surface, thereby exhibiting excellent performance in suppressing Zn dendrites and hydrogen evolution reaction (HER) [39, 40, 41]; (4) K^+^ can contribute additional capacity, the specific capacity of K‐Zn hybrid ion batteries is significantly higher than that of pure zinc ion batteries under the same cathode||anode system [32, 39, 42]; (5) the introduction of larger radius alkali metal cations changes the embedding chemistry of ZnHCF cathode, improving the cathode stability at the expense of capacity [31].

However, most reported high‐performance K‐Zn hybrid systems rely on multi‐variable coupling modification strategies, including cathode doping and specialized electrolyte design [24, 43, 44, 45], which obscure the intrinsic effect of K^+^ on unmodified pure ZnHCF dissolution in dilute aqueous electrolytes. More critically, the potential hazard of localized high K^+^ concentration from current‐driven K^+^ accumulation has been widely overlooked. Specifically, due to a smaller Stokes radius (≈ 3.31 Å) [46, 47] and lower hydration energy [48, 49], trace hydrated K^+^ in K‐Zn mixed electrolyte can diffuse to the cathode surface faster than hydrated Zn^2+^ (≈ 4.30 Å) [50, 51] under current driving, forming a local K^+^‐rich layer. Furthermore, the higher the externally applied current, the faster the K^+^ migrates in the electrolyte inside the battery [52, 53, 54], resulting in a much higher concentration of K^+^ aggregated in the critical K^+^‐rich layer on the cathode surface. This rapid aggregation effect may trigger the dissolution threshold of ZnHCF cathode [55, 56, 57, 58]. Thus, there should be a specific K^+^ concentration in the K‐Zn mixed electrolyte, which ensures the ZnHCF lattice is protected from dissolution caused by excessive K^+^ enrichment under current driving. Therefore, precisely controlling the K^+^ concentration at this specific value is highly instructive, which maximizes the positive effect of K^+^ while ensuring the ZnHCF lattice is not dissolved due to excessive K^+^ enrichment. However, reported K‐Zn mixed electrolyte optimization works only to screen a single static optimal K^+^ concentration under a specific current density, which cannot adapt to the dynamic K^+^ enrichment behavior at the cathode interface regulated by varying current densities. The quantitative criterion for determining the precise K^+^ addition amount under different working current densities has not yet been established.

Herein, the current‐dependent critical dissolution concentration (CDC) is proposed as a reference to determine the optimal K^+^ ratio in the K‐Zn mixed electrolyte, thus achieving stable high‐capacity AZIBs (ZnHCF||Zn) at each current density. Thermodynamically, ZnHCF cathode spontaneously dissolves in a high K^+^ concentration environment, which is essentially driven by the regulation of the reaction quotient *Q* in the *Van't Hoff Isotherm equation*. Combined with the current‐free ZnHCF soaking experiments, the current‐free CDC is obtained. Further, scanning electron microscopy (SEM) and transmission electron microscopy (TEM) confirm that the K^+^ concentration lower than the current‐free CDC also triggers the same ZnHCF dissolution phenomenon, which is attributed to the diffusion effect caused by current. Based on this, high‐resolution transmission electron microscopy (HRTEM) and x‐ray diffraction (XRD) tests of cycled ZnHCF quantify the critical K^+^ concentration corresponding to the optimal ZnHCF lattice structure under gradient current densities. The critical K^+^ concentration corresponds to the electrodeposition/dissolution equilibrium of trace K^+^ support/excess K^+^ dissolution on the ZnHCF cathode surface. Combined with the long‐cycle performance of ZnHCF||Zn batteries at the same gradient current densities, the current density‐CDC curve is fitted. Based on this fitting curve, a specific K^+^ concentration corresponding to a specified current density is successfully predicted and verified with the best electrochemical performance, exceeding other K^+^ concentrations at this current density. In addition, the modulating effect of trace K^+^ on the Zn anode has also been verified.

## 2 Results and Discussion

**FIGURE 1:**
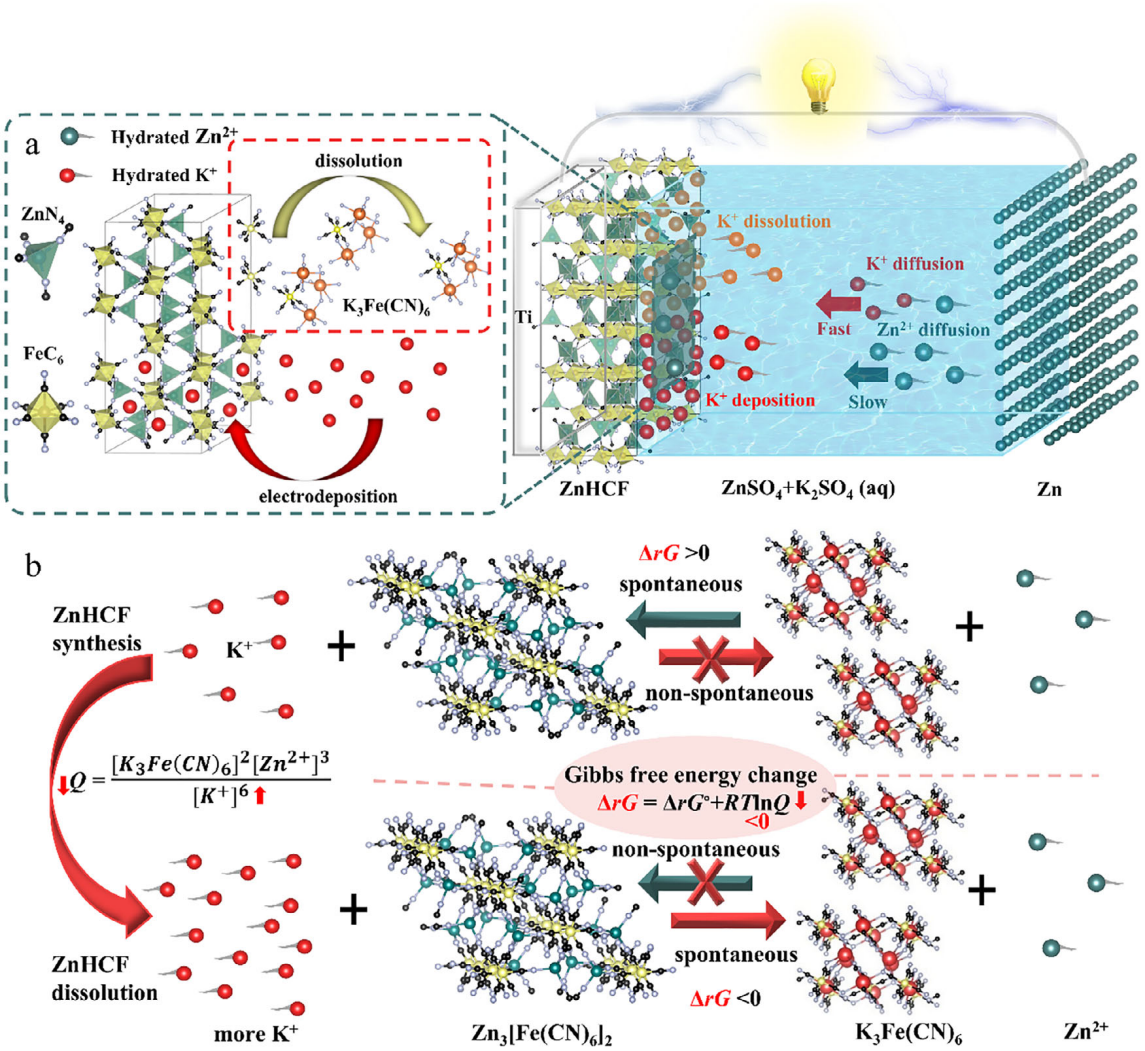
Schematic illustration of (a) the equilibrium between trace K^+^ support/excess K^+^ dissolution in ZnHCF||Zn battery and (b) the reversible ion exchange reactions dominated by K^+^ concentration.

The introduction of trace K^+^ as electrolyte additive in ZnHCF||Zn batteries has been proven an effective approach to contribute extra capacity and support ZnHCF lattice [24, 31, 32]. However, the current‐driven effect causes the cations (K^+^/Zn^2+^) in the mixed electrolyte to migrate toward the ZnHCF cathode, inducing a local inhomogeneous K^+^ distribution (Figure 1a). A large amount of K^+^ is enriched on ZnHCF surface due to faster diffusion rate [53], which disrupts the interaction between Zn^2+^ and coordinated ─N, weakening the Fe─C≡N─Zn bonding and generating soluble K_3_Fe(CN)_6_ [59]. This process is essentially a reversible ion exchange reaction (Figure 1b), and its forward dissolution reaction formula is as follows:

(1) $$\begin{array}{ccc} \mathrm{Zn}3\left[\mathrm{Fe}\left(\mathrm{CN}\right)6\right]2\;\left(s\right) & + & 6K+\left(\mathrm{aq}\right)⇌2K3\mathrm{Fe}\left(\mathrm{CN}\right)6\;\left(\mathrm{aq}\right)\\ & & +3\mathrm{Zn}2+\left(\mathrm{aq}\right) \end{array}$$

where Zn3[Fe(CN)6]2 (s) is solid zinc hexacyanoferrate (ZnHCF); K^+^ (aq), K_3_Fe(CN)_6_ (aq), and Zn^2+^ (aq) are potassium ions, soluble ferricyanide, and zinc ions in aqueous solution, respectively; “⇌” indicates that the reaction is reversible. Further, the spontaneity of the reaction in solution is determined by the Gibbs free energy change (Δ*rG*) in *Van't Hoff Isotherm equation* [60], which is expressed quantitatively as follows:

(2) $$\Delta \mathrm{rG}\;=\Delta {\mathrm{rG}}^{\circ }+\mathrm{RTlnQ}$$

where Δ*rG* is the Gibbs free energy change of the reaction under any conditions, in kJ/mol; Δ*rG°* is the Gibbs free energy change of the reaction under standard conditions which refer to 298 K, 100 kPa, and each solute concentration of 1 mol·L^−1^, in kJ·mol^−1^; *R* is the ideal gas constant 8.314 J·(mol·K)^−1^; *T* is the absolute temperature (in K); *Q* is the reaction quotient, reflecting the concentration relationship between reactants and products at any given time.

For the above ion exchange reaction, the calculation of the reaction quotient *Q* must follow the rule that the concentration of the solid substance is not included in the expression [61], thus the revised result is as follows:

(3) $$Q=\frac{{\left[K_{3}\mathrm{Fe}{\left(\mathrm{CN}\right)}_{6}\right]}^{2}{\left[Zn^{2+}\right]}^{3}}{{\left[K^{+}\right]}^{6}}$$

where [*K_3_Fe(CN)_6_*], [*Zn^2+^*], and [*K^+^*] represent the concentrations of K_3_Fe(CN)_6_, Zn^2+^, and K^+^ in the aqueous solution, respectively, in mol·L^−1^; the exponents correspond to the stoichiometric coefficients of each substance in the reaction equation.

Generally, ZnHCF is spontaneously synthesized by the reaction of Zn^2+^ with a low concentration of K_3_Fe(CN)_6_ at room temperature [31, 59, 62, 63, 64, 65]. According to the second law of thermodynamics [66], the Gibbs free energy of spontaneous ZnHCF synthesis is negative (Δ*rG* <0). Conversely, the reverse reaction (ZnHCF dissolution) cannot proceed spontaneously with low K^+^ concentration at room temperature (Figure 1b); the corresponding Gibbs free energy is positive (Δ*rG* >0). However, the high concentration of K^+^ enriched on the ZnHCF surface disrupts this thermodynamic equilibrium, making the potentially non‐spontaneous ZnHCF dissolution reaction spontaneous (Figure 1b): according to *Le Chatelier's principle* [67], an increase in [*K^+^*] will drastically change the value of the reaction quotient *Q* (an increase in the denominator leads to a significant decrease in *Q*). When the K^+^ concentration is sufficiently high, and *Q* drops to a critical value, the Δ*rG* of the entire reaction will satisfy the spontaneous condition of Δ*rG* <0, thus allowing the originally non‐spontaneous ZnHCF dissolution reaction to occur spontaneously. Macroscopically, this is manifested as the dissolution of ZnHCF in a high‐concentration K^+^ environment, generating soluble K_3_Fe(CN)_6_. The contradiction between “ZnHCF spontaneous synthesis under low K^+^” and “ZnHCF spontaneous dissolution under high K^+^” stems from the regulatory effect of K^+^ concentration on the reaction quotient *Q*, which directly reflects the concentration dependence of K^+^ on the stability of ZnHCF.

**FIGURE 2:**
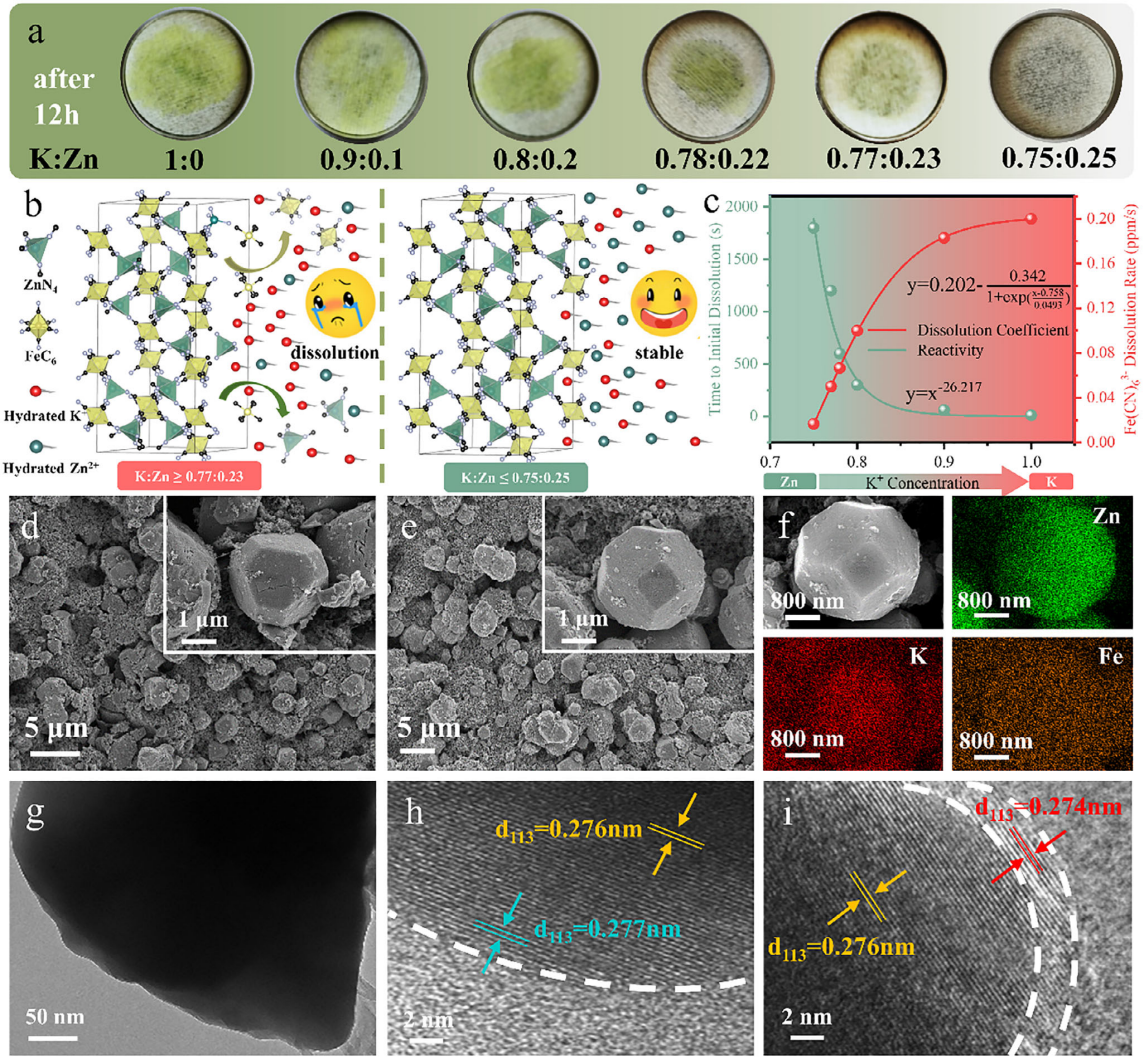
(a) Color change after adding K‐Zn mixed solutions onto ZnHCF surface for 12 h without current. (b) Schematic diagram of ZnHCF lattice dissolution in high K^+^ ratio K‐Zn mixed solutions. (c) Quantitative schematic of ZnHCF lattice reactivity and dissolution rate in K‐Zn mixed solutions with fitted kinetic models. SEM images of cycled ZnHCF cathode using (d) 0.2K electrolyte and (e) 0.1K electrolyte. (f) Elements distribution of cycled ZnHCF cathode using 0.1K electrolyte. (g) TEM images of cycled ZnHCF cathode using 0.1K electrolyte. HRTEM images of cycled ZnHCF cathode using (h) 0.1K electrolyte and (i) 0.2K electrolyte.

To quantify the regulatory effect of high‐concentration K^+^ on the spontaneity of ion exchange reactions of ZnHCF by adjusting the reaction quotient *Q*, a current‐free ZnHCF soaking experiment is first performed from an intrinsic chemical perspective. ZnSO_4_/K_2_SO_4_ mixed solution with a total cation concentration of 1 m is prepared, with the K:Zn molar ratio systematically adjusted as: 1:0 (1K), 0.9:0.1 (0.9K), 0.8:0.2 (0.8K), 0.78:0.22 (0.78K), 0.77:0.23 (0.77K), and 0.75:0.25 (0.75K). A series of K‐Zn mixed solutions is then dropped onto the glass microfiber separator pre‐coated on ZnHCF and stand for 12 h to observe the discoloration caused by ZnHCF dissolution (Figure 2a). Due to the inherent ligand‐to‐metal charge transfer [68, 69, 70] of the dissolved Fe(CN)_6_^3−^, the ZnHCF dissolved in a high K^+^ environment appears yellow‐green. The more it dissolves, the greener the color becomes. Figure 2a reveals samples with a K:Zn ratio ≥0.77:0.23 (marked as 0.77 K) change significantly from colorless to light yellow–green, while samples with a K:Zn ratio ≤0.75:0.25 (marked as 0.75K) remain colorless. This visible color change suggests ZnHCF lattice dissolution at high K^+^ ratios in the mixed solution. Correspondingly, when [*K^+^*] ≥0.77K, Δ*rG* <0, and the ZnHCF dissolution reaction proceeds spontaneously; when [*K^+^*] ≤0.75K, Δ*rG* >0, and ZnHCF remains stable (Figure 2b).

To further quantitatively analyze the degree of dissolution, inductively coupled plasma optical emission spectrometry (ICP‐OES) is used to detect the contents of Zn/Fe elements, which are from the dissolved ZnHCF. Unsoaked 1K solution demonstrates 0.001 ppm of Zn and Fe elements (instrument background level), while the 1K solution with ZnHCF soaked for 12 h demonstrate significant high Zn and Fe elements contents (Figure S1). High Zn and Fe elements in 1K solution confirm the ZnHCF dissolution. Correspondingly, x‐ray diffraction (XRD) patterns of soaked and unsoaked ZnHCF (Figure S2) also give evidence of ZnHCF dissolution: the peak intensity of ZnHCF (113) crystal plane decreases significantly after soaking in 1K solution for 12 h, accompanied by a slight broadening, indicating ZnHCF lattice distortion.

The time to initially observe the color change with different K^+^ ratios is also recorded to reflect the reactivity. As shown in the cyan curve of Figure 2c, the time to observe the initial change of K:Zn = 0.77:0.23 (0.77K) is more than 1200 s, while that of K:Zn = 1:0 (1K) is less than 10s. Notably, no significant discoloration is observed for the sample corresponding to K:Zn = 0.75:0.25 (0.75K) after more than 1800s, which verifies the regulation of K^+^ concentration on ZnHCF reactivity. The reactivity curve is quantitatively fitted via a power function model, with the fitting formula of *y*  = *x* ^−26.217^ , where *y* is the time to initial dissolution (s), and *x* is the molar ratio of K^+^ in the electrolyte. Furthermore, to demonstrate the dissolution rate, the increased value of ion concentration in 10 min soaking experiments is also tested and calculated via ICP‐OES (Figure S3), which shows that the dissolved Zn and Fe keep a synchronous growth trend with the proportion of K^+^ in the mixed solution. Based on this, the dissolution coefficient is calculated as the red curve (Figure 2c). The release rate of Fe(CN)_6_^3−^ increases significantly with increasing K^+^ concentration, finally reaching 0.20 ppm/s at K:Zn = 1:0 (1K). The dissolution coefficient curve is quantitatively fitted via a Boltzmann sigmoidal model, with the fitting formula of $y=0.202-\frac{0.342}{1+e^{\left(\frac{x-0.758}{0.0493}\right)}}$, where *y* is the dissolution rate of Fe(CN)_6_^3−^ (ppm/s) and *x* is the molar ratio of K^+^ in the electrolyte. This demonstrates that a high K^+^ ratio can significantly accelerate the lattice collapse of ZnHCF even in an extremely short time.

Based on the above soaking experiments under static conditions with no current influence, K^+^ in the mixed solution will cause the dissolution and structural damage of ZnHCF, and the higher the K^+^ concentration, the more obvious the dissolution phenomenon. High K^+^ concentration (>0.75K) triggers significant chemical dissolution of the ZnHCF lattice, which corresponds to the critical concentration [*K^+^*] determining the spontaneous direction of the ion exchange reaction. It should be noted that this critical dissolution concentration of K:Zn cannot be directly extrapolated to charge–discharge processes. Under actual electrochemical conditions, electric field‐driven ion migration will further alter the local concentration distribution at the cathode‐electrolyte interface [52], potentially triggering similar dissolution phenomena at even lower overall K^+^ concentrations. The current‐free critical dissolution concentration of K:Zn provides an essential reference for subsequent current‐dependent experiments.

To verify the specific K^+^ concentration triggering ZnHCF lattice dissolution under work conditions, the current‐dependent experiment is conducted. ZnHCF||Zn batteries using electrolytes with three different ratios (0.2 K + 0.8 Zn, 0.1 K + 0.9 Zn, and 0.05 K + 0.95 Zn, marked as 0.2K, 0.1K, and 0.05K, respectively) are assembled and cycled at 250 mA·g^−1^. The SEM images and element distribution of the ZnHCF cathode with different electrolytes after 20 cycles are displayed in Figure 2d–f and Figure S4. Figure 2d and Figure S4b show the surface of the ZnHCF cathode using 0.2K electrolyte and the visible cracks accompanied by local collapse, indicating the significant degradation of the ZnHCF cathode. In contrast, that of the 0.1K sample remains intact, with only localized microcracks (Figure 2e and Figure S4c) corresponding to less degradation. The morphology of the ZnHCF using 0.05K electrolyte is similar to 0.1K, with only occasional minor surface defects (Figure S4a,d). The XRD comparison between the pristine ZnHCF and cycled samples further provides quantitative evidence for this trend (Figure S5): taking the peak intensity of the characteristic (113) plane of pristine ZnHCF as the benchmark, the 0.1K sample exhibits the best peak intensity retention after cycling, while the 0.2K sample shows significant peak intensity attenuation, confirming the severe structural degradation of the cathode in high K^+^ electrolyte. These results show a similar trend to the current‐free ZnHCF dissolution phenomenon: higher K^+^ concentration (0.2K) causes stronger ZnHCF structural damage. It is worth noting that the current‐free critical dissolution concentration (marked as CDC, ≈0.75K) is inconsistent with the current‐dependent critical dissolution concentration (CDC≈0.1–0.2K) at 250 mA·g^−1^. The current‐dependent CDC (0.1–0.2K) is smaller because K^+^ is enriched under current driving, so a lower K^+^ content is required in K‐Zn mixed electrolyte to trigger the dissolution and structural destruction of ZnHCF.

Energy dispersive spectrometry (EDS) further reveals the current‐dependent structural damage caused by K^+^. As shown in Figure 2f and Figure S6, the Zn, K, Fe, C, and N elements are uniformly distributed. Further, Table S1 systematically measures and compares the content of various elements in ZnHCF cycled with different K^+^ ratio electrolytes after 20 times at 250 mA·g^−1^: the Zn and Fe atomic content of the 0.2K sample is lowest, while that of the 0.1K and 0.05K samples is higher. Since the ZnHCF framework is composed of ZnN_4_ tetrahedra and FeC_6_ octahedra [62], lower Zn and Fe contents of 0.2K sample confirm that higher K^+^ concentration causes stronger ZnHCF structural damage. The supplementary ICP‐OES test of the cycled cathodes, as shown in Table S2, further accurately confirms that higher K^+^ concentration leads to more severe structural damage of ZnHCF.

TEM images are shown in Figure 2g–i and Figure S7. The HRTEM image of ZnHCF electrode using 0.1K electrolyte in Figure 2h illustrates that the edge layer distance is 0.277 nm, slightly larger than the 0.276 nm within the lattice. Wider edge layer distance suggests a slight lattice expansion, indicating that trace K^+^ insertion can widen the ion channel without triggering dissolution. The supplementary TEM‐energy dispersive x‐ray spectroscopy (EDS) mapping results in Figure S8 further confirm the uniform distribution of K element in the cycled ZnHCF lattice, providing direct elemental evidence for the intercalation of K^+^ into the ZnHCF framework. In contrast, the edge layer distance of the ZnHCF electrode using 0.2K electrolyte is only 0.274 nm, lower than the 0.276 nm within the lattice (Figure 2i). The narrower edge layer distance suggests surface lattice contraction. These results are consistent with the XRD pattern in Figure S2; the θ value of the (113) crystal plane increases, confirming the reduction of layer distance. The change of layer distance is caused by: (1) the radius of hydrated K^+^ (3.3 Å) better matches the lattice channel size of typical Prussian blue analogs (3.2–4.6 Å) [71, 72, 73, 74] and its hydration energy is lower, thus K^+^ is more prone [31, 32, 45] to undergo dehydration before intercalation into the ZnHCF lattice channel compared to hydrated Zn^2+^ (4.3 Å); (2) before reaching the critical dissolution concentration (CDC), the higher the K^+^ concentration, the more effective K^+^ insertion, the more significant the corresponding filling effect, which lead the greater degree of lattice expansion as the insertion of K^+^ expand the layer distance; (3) when the critical dissolution concentration (CDC) is exceeded, excess K^+^ reduces the reaction quotient *Q*, making the ion exchange reaction spontaneous (Δ*rG* <0). The spontaneous ion exchange reaction leads to the dissolution of [Fe(CN)_6_]^3−^ which causes ZnHCF lattice collapse, as shown by TEM observations of decreased edge layer distance and a rightward shift of the XRD peak toward higher 2θ angles.

Compared to the current‐free critical dissolution concentration (≈0.75K), a lower K^+^ ratio (0.1–0.2K) triggers ZnHCF cathode dissolution under current influence. This is because the electric field drives K^+^ enrichment at the cathode‐electrolyte interface, making the interfacial localized K^+^ concentration much higher than the bulk K^+^ concentration. Taking 250 mA·g^−1^ as a representative example, the TEM characterization results strongly suggest that the interfacial localized K^+^ concentration for the 0.1K electrolyte should be close to the 0.75 m static threshold without exceeding it, while the interfacial localized K^+^ concentration for the 0.2K electrolyte under the same current density has exceeded this threshold, leading to cathode dissolution. In addition, it is clear that the insertion support and induced dissolution of K^+^ both occur in the ZnHCF lattice edge, and the (113) crystal plane is particularly sensitive to the insertion/extraction of K^+^.

**FIGURE 3:**
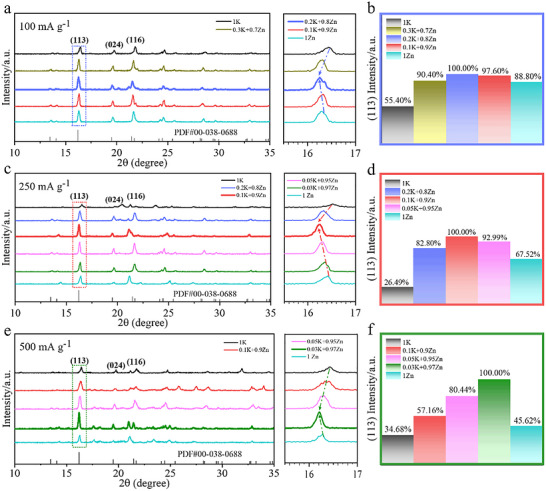
XRD patterns of ZnHCF cathode cycled at (a) 100, (c) 250, and (e) 500 mA·g^−1^. The (113) peak intensity of ZnHCF cathode cycled at (b) 100, (d) 250, and (f) 500 mA·g^−1^.

To systematically reveal the relationship between the ZnHCF (113) crystal plane and K^+^ insertion/extraction, ZnHCF||Zn full batteries are assembled using a series of K‐Zn mixed electrolytes (total cation concentration 1 m) at current densities of 100, 250, and 500 mA·g^−1^ and the x‐ray diffraction (XRD) analysis of ZnHCF cathode is further carried out (Figure 3). The (113) crystal plane is not only the core active plane for K^+^ intercalation regulation, but also the strongest diffraction peak of the rhombohedral ZnHCF phase with the highest signal‐to‐noise ratio, which can stably and quantitatively reflect the bulk lattice evolution of the material. Thus, the peak positions and intensities of the ZnHCF (113) crystal plane after 50 cycles are systematically compared as the core analytical object.

As shown in Figure 3, the 2θ value of the (113) plane shows a characteristic “first left then right” shift with the increase of K^+^ content in the electrolyte at any current density. Taking the 1 Zn sample (also marked as 0K) in 100 mA·g^−1^ XRD patterns as a benchmark (Figure 3a), as the K^+^ concentration increases, the peak position movement of (113) plane, which first shifts leftward to the limit at 0.2 K + 0.8 Zn (marked as 0.2K, Figure 3a, blue line) and then turns right, outlines a typical “**<**” shaped curve. The same trend is observed in 250 and 500 mA·g^−1^ XRD patterns, with the peak position of (113) plane shifting to the left‐most at 0.1 K + 0.9 Zn (marked as 0.1K, Figure 3d, red line) and 0.03 K + 0.97 Zn (marked as 0.03K, Figure 3f, green line). The K^+^ concentration corresponding to the left‐most part of the “**<**” shaped curve at each current density is the critical dissolution concentration (CDC) under this condition.

The (116) crystal plane is also a characteristic diffraction peak of rhombohedral ZnHCF. This crystal plane is further analyzed to verify the universality of the K^+^‐regulated lattice evolution law. Across all tested current densities, the (116) peak exhibits the same characteristic “<” shaped shift trend with the increase of K^+^ content in the electrolyte, confirming that the lattice strain regulation effect of K^+^ on ZnHCF is not limited to a single crystal plane. It should be noted that all XRD tests in this work are ex situ characterizations performed on independent ZnHCF electrodes disassembled after cycling. The low‐intensity secondary diffraction peaks are more susceptible to the inherent differences in local crystallinity and interfacial structural heterogeneity between different post‐cycle electrodes, resulting in slight and normal fluctuations in their peak shift amplitude, while the overall change trend remains consistent with the (113) crystal plane that reflects the bulk lattice properties.

According to the *Bragg equation 2dsinθ = nλ* [75], a decreased 2θ value indicates lattice expansion. Conversely, an increase in 2θ indicates lattice contraction. Therefore, this “**<**” shaped peak shift of (113) plane reflects the competitive lattice strain regulated by K^+^ concentration: (1) when the K^+^ ratio gradually increases from an extremely low percentage, trace hydrated K^+^ in the electrolyte will undergo dehydration before intercalating into the ZnHCF lattice channel and larger K^+^ radius causes lattice expansion and the 2θ shifts to the left [31, 45]; (2) when the K^+^ ratio continues to increase over critical dissolution concentration (CDC), excess K^+^ forms a K^+^‐rich layer under current driving, high concentration of K^+^ promote the spontaneous ZnHCF dissolution by altering the reaction quotient Q, thus local ZnHCF framework becomes unstable and collapses, resulting in “d” value decrease and a subsequent 2θ rightward shift; (3) only when the K^+^ ratio just matches critical dissolution concentration (CDC) can the K^+^‐rich layer balance the lattice expansion of trace K^+^ and the lattice dissolution of excess K^+^. Taking 250 mA·g^−1^ XRD patterns as an example (Figure 3c), 0.1K + 0.9 Zn (marked as 0.1K, red line) is exactly the current‐dependent critical concentration verified by the aforementioned SEM/TEM results, where trace K^+^ support and excess K^+^ dissolution reach equilibrium.

In addition to the “**<**” shaped peak position shift, the peak intensity of the (113) plane also shows a typical fluctuation. Taking 100 mA·g^−1^ XRD patterns as an example (Figure 3b), the highest peak intensity of the (113) plane for the 0.2K sample (blue column) is set as 100%. Regardless of whether the K^+^ ratio is lower or higher than 0.2K, the peak intensity of the (113) plane decreases significantly. The same trend is repeated under 250 and 500 mA·g^−1^, with the highest peak intensities observed at 0.1K (Figure 3d, red column) and 0.03K (Figure 3f, green column). This intensity fluctuation is attributed to the K^+^ concentration‐dependent regulation of lattice order: appropriate trace K^+^ insertion supports the ZnHCF lattice structure and improves the cathode material stability [31]. This ensures minimal exfoliation of the electrode active material after charge–discharge cycling, resulting in the highest peak intensity. However, when the K^+^ ratio is too high, the current‐driven formed K^+^‐rich layer weakens the polarization of the Fe─C≡N─Zn bond, leading to Fe(CN)_6_^3−^ dissolution and the increase of local cracks/pores (Figure S4b). This reduces the coherent diffraction area, similarly causing peak intensity attenuation and broadening.

It should be clarified that the K^+^ concentration in the electrolyte is the fundamental trigger for the lattice structure evolution, while the altered interlayer spacing induced by K^+^ intercalation is the key intermediate structural process that directly determines the lattice integrity and dissolution behavior of the ZnHCF cathode. Notably, at all three current densities, the K:Zn ratios corresponding to the peak position shifts and intensity fluctuations are consistent, indicating the same structural regulation mechanism. Furthermore, it is noted that as current density increases, the overall K^+^ concentration required to achieve localized K^+^ enrichment (also the CDC in cathode‐electrolyte interface) shows a decreasing trend (0.2→0.1→0.03K), suggesting that the coupling of ion migration and external current reduces the critical K^+^ requirement for the K^+^‐rich layer formation.

**FIGURE 4:**
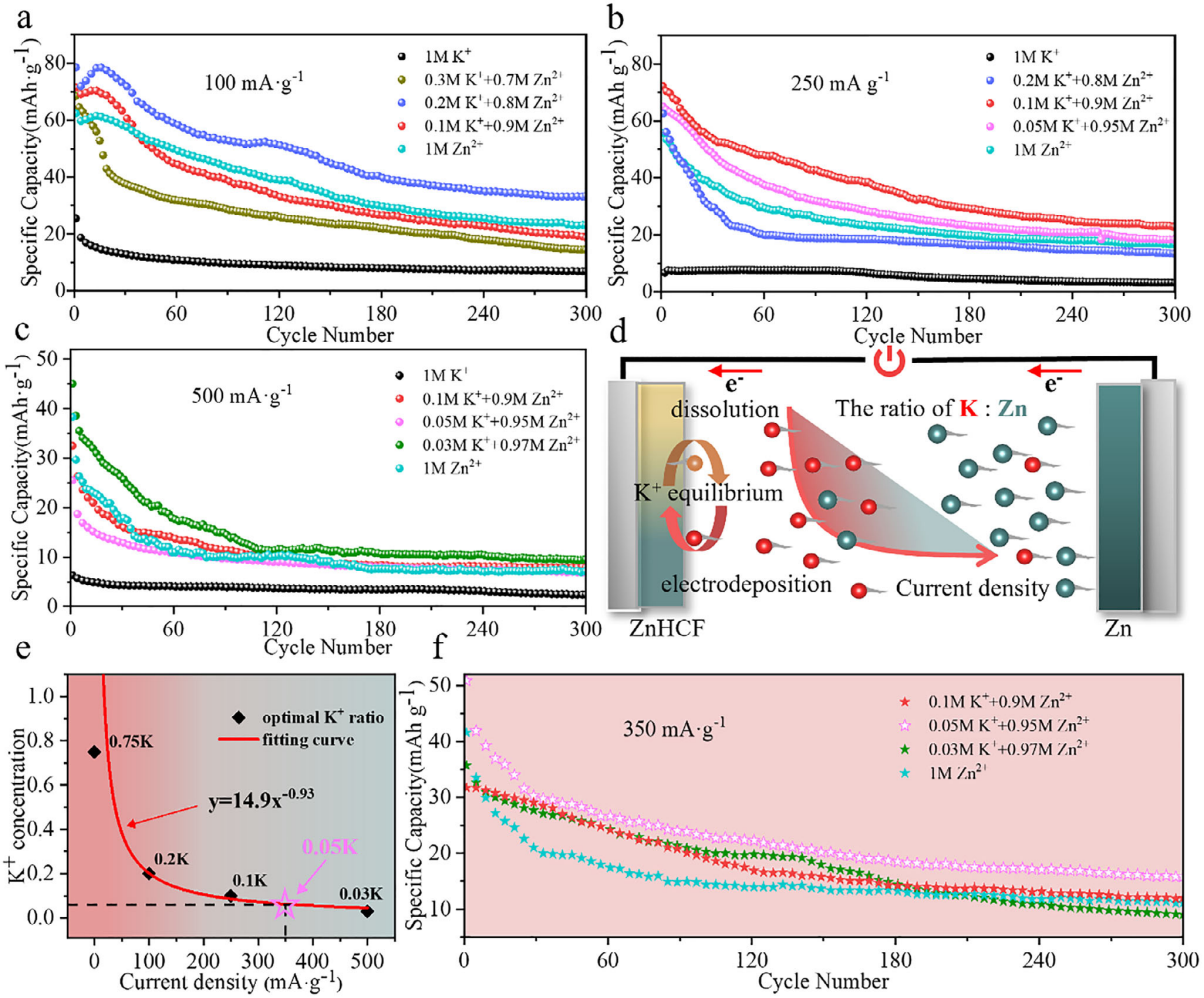
Long cycle performance at (a) 100 mA·g^−1^, (b) 250 mA·g^−1^, and (c) 500 mA·g^−1^. (d) The schematic diagram of current‐dependent K^+^ equilibrium of electrodeposition/dissolution on ZnHCF cathode surface. (e) A fitted curve quantifying the relationship between current density and critical dissolution concentration. (f) Long cycle performance at 350 mA·g^−1^.

To verify the coupling regulation of ion migration and external current on ZnHCF cathode, ZnHCF||Zn full batteries are tested for long‐term cycling using K‐Zn mixed electrolytes with the corresponding ratios in XRD patterns. As shown in Figure 4a, the ZnHCF||Zn full battery with 0.2K electrolyte (blue) exhibits the highest initial specific capacity of 81 at 100 mA·g^−1^. Compared with higher or lower K^+^ concentration, the overall capacity of the 0.2K sample shows a significant advantage, fully confirming the prediction of position shift and intensity fluctuation of the (113) plane in Figure 3a,b. When the current density increases to 250 and 500 mA·g^−1^, the K/Zn concentration with the highest overall capacity shifts to 0.1K (red, Figure 4b) and 0.03K (green, Figure 4c), and exhibits the same patterns as 100 mA·g^−1^ (Figure S9). This confirms the equilibrium between trace K^+^ support and excess K^+^ dissolution shown in the XRD patterns. Taking 250 mA·g^−1^ as an example (Figure 4b), the overall specific capacity of the red 0.1K sample is significantly higher than other ratios. Therefore, taking 0.1K as the dividing line, the relatively higher K^+^ ratio and the lower K^+^ ratio are divided for separate discussion.

For relatively higher K^+^ ratio: compared with the 0.1K sample (red), the initial specific capacity of the 0.2K sample (blue) is only 62 mAh·g^−1^ (Figure 4b). This is due to the excessive enrichment of trace K^+^ on the ZnHCF surface under current driving, which induces ZnHCF cathode dissolution. The loss of cathode active material (ZnHCF) reduces the specific capacity of the ZnHCF||Zn full battery, corresponding to the decrease in peak intensity of the (113) plane (blue column, Figure 3d). Correspondingly, Figure S10a–c reveals that higher K^+^ concentration results in lower initial coulombic efficiency (CE), which stems from irreversible side reactions and active material loss during the first charge. This phenomenon is limited to the initial cycle, and K^+^ within the critical dissolution concentration range enables rapid CE recovery and significantly enhanced long‐term cycling stability. Notably, the 1K sample (black), with the extremely highest K^+^ concentration, exhibits the lowest initial specific capacity of only 7 mAh·g^−1^ (Figure 4b). This indicates the ZnHCF cathode suffered severe K^+^‐induced dissolution during the rest period (12 h) after battery assembly. Figure S10a–c shows that the initial CE of the 1K sample (black) is less than 70%. Although it gradually recovers to approximately 90% within 300 cycles, significant loss of ZnHCF has already occurred, resulting in a remaining specific capacity of only 3 mAh·g^−1^ after 300 cycles.

For relatively lower K^+^ ratio: compared to 0.1K sample (red), the initial specific capacity of the 0.05K sample (pink, Figure 4b) also drops from 71 mAh·g^−1^ (0.1K) to 65 mAh·g^−1^. This is due to the lack of additional capacity contribution from K^+^ [42]. Besides the lack of additional capacity, insufficient K^+^ also impacts long‐term cycling stability. Particularly, the 1 Zn sample (cyan), devoid of any K^+^, has an initial CE of 105%, which is higher than other K^+^ containing ratios. However, after 300 cycles, its specific capacity remains at only 16 mAh·g^−1^ with capacity retention of 29.1% (Figure 4b). This suggests that exclusive Zn^2+^ insertion also leads to cathode structural distortion, resulting in a sharp decline in capacity [31]. Appropriate K^+^ insertion can support ZnHCF lattice channels and improve the cathode stability.

For the critical dissolution concentration (CDC): 0.2K (100 mA·g^−1^, Figure 4a), 0.1K (250 mA·g^−1^, Figure 4b), and 0.03K (500 mA·g^−1^, Figure 4c) are all equilibrium threshold points at various current densities. The K/Zn ratio at this critical point balances both capacity and stability. Other ratios either exacerbate dissolution due to excessive K^+^ or lack additional capacity contribution due to insufficient K^+^. This performance balance is intrinsically rooted in the lattice evolution revealed by Figure 3: K^+^ matching the CDC maintains moderate interlayer spacing and lattice integrity, ensuring sufficient capacity output and excellent cycling stability.

On this basis, it is noted that in the ZnHCF||Zn batteries, the greater the current density, the lower the critical dissolution concentration (CDC) (Figure 4d). This is because hydrated K^+^ diffuses more rapidly at high currents owing to its smaller Stokes radius [53]. Consequently, the bulk K^+^ content required to reach the critical dissolution concentration (CDC) at the ZnHCF surface decreases. This avoids excessive K^+^ accumulation that can induce spontaneous ZnHCF lattice dissolution, maximizing the stabilizing effect and additional capacity contribution of K^+^.

To further quantify the relationship between current density and critical dissolution concentration (CDC), a fitting curve (*y* = 14.9*x* ^−0.93^) is plotted as a criterion to predict the optimal K^+^ concentration at any current density, with current density as the *x*‐axis and K^+^ concentration as the *y*‐axis. As shown in Figure 4e, this curve closely matches the four points: 0.75K (current‐free, Figure 2b), 0.2K (100 mA·g^−1^, Figure 4a), 0.1K (250 mA·g^−1^, Figure 4b), and 0.03K (500 mA·g^−1^, Figure 4c). To verify the reliability of the fitting curve, a pink star with a current density *x* = 350 mA·g^−1^ and the K^+^ concentration *y* = 0.05K is selected for long‐term cycling verification. As shown in Figure 4f, the pink sample (0.05 K + 0.95 Zn, marked as 0.05K) exhibits an initial specific capacity of 47 mAh·g^−1^, exceeding that of other K:Zn ratios samples at 350 mA·g^−1^. This further validates the reliability of this fitting curve for predicting the optimal K^+^ ratio at different current densities.

To verify the universality of the proposed CDC criterion, supplementary verification experiments are performed using a carbon‐composited ZnHCF (ZnHCF@C) cathode with enhanced intrinsic structural stability. At 500 mA·g^−1^, the optimal K^+^ concentration for the ZnHCF@C cathode shifts to 0.2K (with a capacity retention of 71.2% after 500 cycles), significantly higher than the 0.03K optimal ratio for pristine ZnHCF at the same current density (Figure S11). The core regulation law of K^+^ on cathode stability remains fully consistent with the CDC criterion: appropriate K^+^ addition enhances cycling stability, while excessive K^+^ triggers severe cathode dissolution and performance degradation. This result confirms that the critical dissolution concentration of K^+^ is closely related to the intrinsic structural stability of the cathode material, demonstrating the universal guiding value of our CDC criterion for electrolyte optimization of ZnHCF‐based cathodes with different stability.

Figure S12a–f further confirms this conclusion from a kinetic perspective: the galvanostatic charge–discharge (GCD) curves exhibit continuous slopes rather than typical flat plateaus, indicating that the competitive insertion of K^+^/Zn^2+^ into ZnHCF is closer to a solid‐solution process (Figure S12a–c). All long‐cycle performance tests were strictly conducted within a safe voltage window of 1.0–2.0 V (below the aqueous electrolyte decomposition potential ≈2.0 V). The first cyclic voltammetry (CV) reveals that the oxidation/reduction peaks in the 1 Zn electrolyte are located at 1.93/1.71 V, with low peak currents (Figure S12d). In contrast, the peaks in the 1K electrolyte (Figure S12e) shift positively to 2.15/1.82 V, and the peak current increases significantly, indicating faster K^+^‐mediated insertion kinetics, but a significant portion of the charge is consumed by side reactions. Furthermore, the first three CV cycles of the 0.1K sample (Figure S12f) only discern a single oxidation peak close to 1K, with two peaks in the reduction direction (≈1.79 and 1.62 V), and a gradually increasing potential gap. This reflects a two‐step insertion pathway, first K^+^ and then Zn^2+^, and a gradual increase in polarization caused by side reactions during the initial cycling phase, which is consistent with the slow recovery of CE and capacity retention during long cycling. Notably, the slightly extended upper voltage limit in CV characterization is only applied for clear redox peak resolution, and CV tests involve merely 3–5 short cycles with negligible electrolyte decomposition, which does not affect redox peak identification or mechanistic analysis.

**FIGURE 5:**
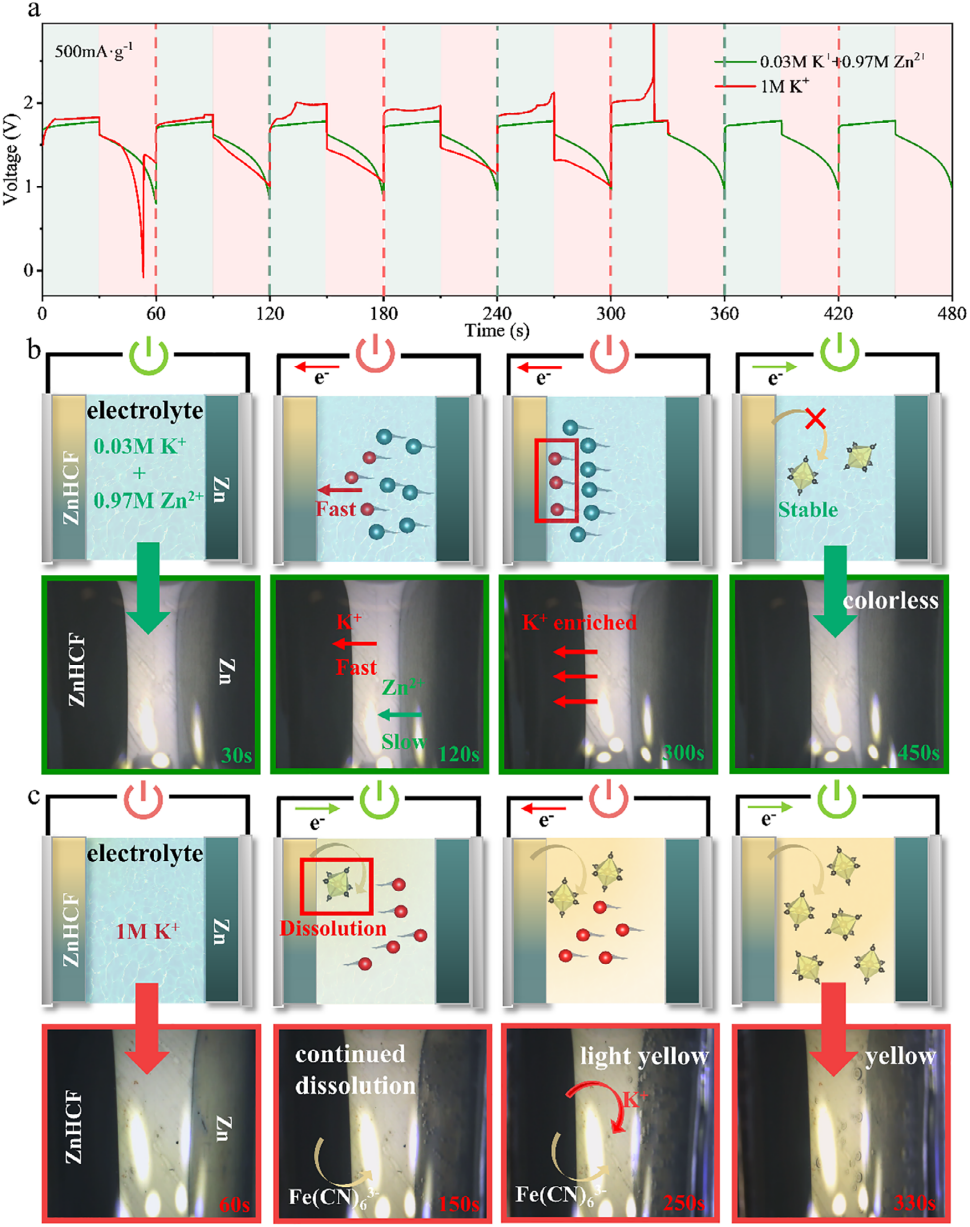
(a) Galvanostatic charge–discharge curves of in situ ZnHCF||Zn full cell using 0.03 and 1K electrolyte at 500 mA·g^−1^. The in situ optical microscopy observation of color change of (b) 0.03K and (c) 1K electrolyte at 500 mA·g^−1^.

To obtain visual evidence of dissolution kinetics at the ZnHCF cathode‐electrolyte interface, an in situ ZnHCF||Zn full cell is designed for optical observation (Figure 5), which verifies the critical dissolution concentration (0.03K) given by long‐term cycling at 500 mA·g^−1^ (Figure 4c). As shown in the GCD curves at 500 mA·g^−1^ (Figure 5a): compared to the 1K system, the 0.03K system exhibits a stable and reproducible voltage plateau. Furthermore, the 0.03K electrolyte remains colorless and transparent during cycling (Figure 5b), while the 1K electrolyte gradually turns yellow at the same current density (Figure 5c). The 1K electrolyte color change indicates ZnHCF structural degradation and continued Fe(CN)_6_^3−^ dissolution at a K^+^ rich environment. Correspondingly, 0.03K is the equilibrium threshold between trace K^+^ support and excess K^+^ dissolution at 500 mA·g^−1^. Consequently, there is virtually no significant ZnHCF dissolution, and the electrolyte naturally exhibits no noticeable discoloration. Additionally, an unconventional in situ ZnHCF||ZnHCF observation device with applied voltage is designed for pure cathode‐electrolyte interface evaluation. This test is performed under extreme conditions only for accelerated verification of the cathode dissolution behavior, and does not represent the conventional working condition of the electrode. As shown in Figure S13, the yellowing phenomenon of the K^+^ rich electrolyte is more pronounced and obvious, further confirming the Fe(CN)_6_^3−^ dissolution from degraded ZnHCF. Further control experiments with other common alkali metal cations (Li^+^ and Na^+^) were conducted to clarify the specificity of the K^+^ regulation effect on the ZnHCF cathode. As shown in Figure S14, both Li^+^ and Na^+^ only bring negligible improvement in cycling performance compared with K^+^. This phenomenon can be attributed to the fact that the physicochemical properties of Li^+^ and Na^+^, including their larger hydrated radius and lower structural pillar effects [31, 45], fail to match the optimal lattice regulation requirements of unmodified pure ZnHCF as effectively as K^+^.

**FIGURE 6:**
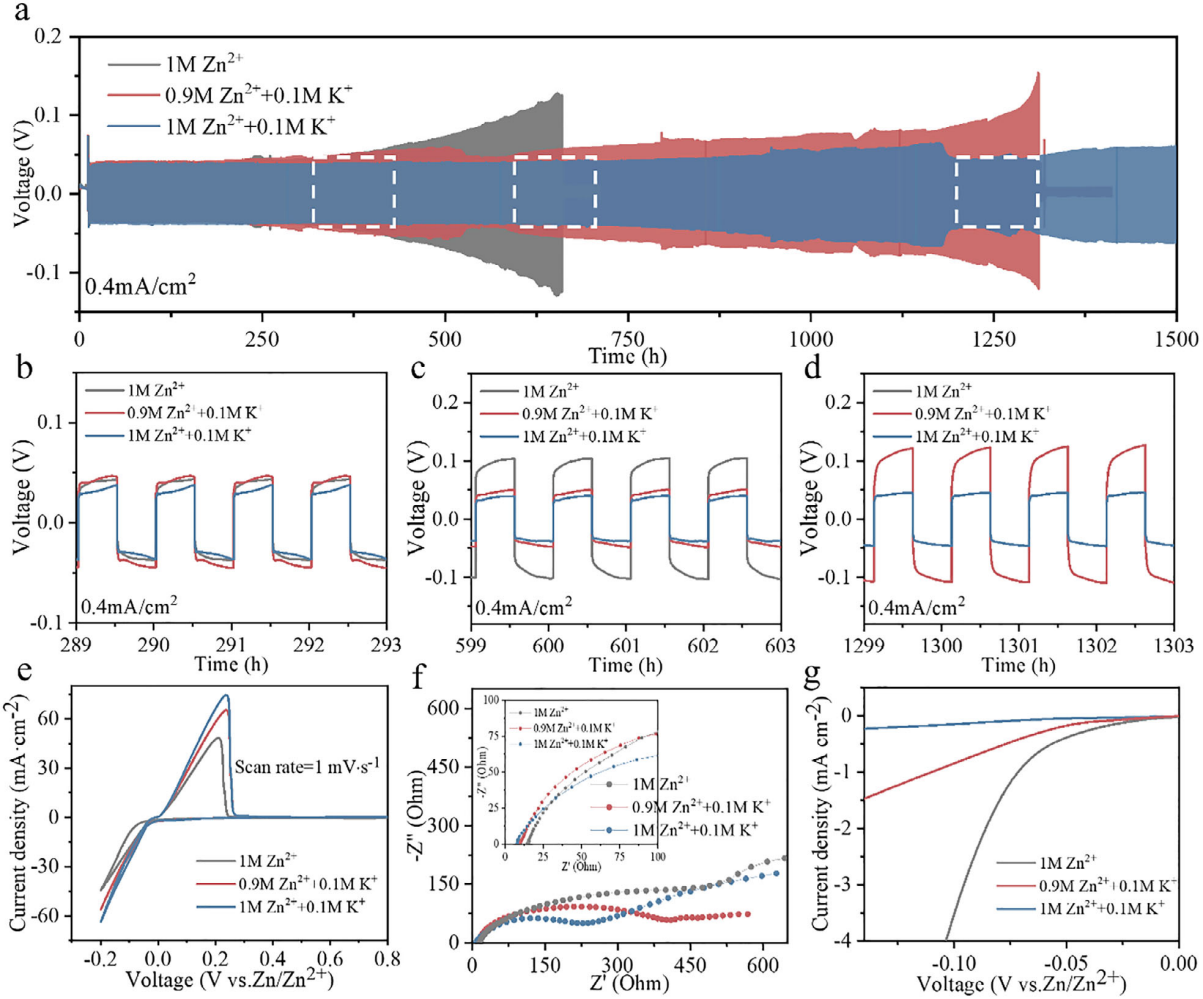
(a) Galvanostatic test of Zn||Zn symmetric cells with different electrolytes at 0.4 mA·cm^−2^ and 0.2 mAh·cm^−2^. The detail magnification image of galvanostatic test of Zn||Zn symmetric cells at about (b) 300, (c) 600, and (d) 1300 cycles. (e) CV profiles of the Zn||Cu asymmetric cells with a scan rate of 1 mV·s^−1^ in different electrolytes. (f) EIS of Zn||Zn symmetric cells at an open‐circuit voltage in different electrolytes. (g) LSV of the Zn anode at 1mV·s^−1^ in different electrolytes.

In addition to the dissolution‐electrodeposition equilibrium induced on the ZnHCF cathode surface, the introduction of K^+^ also affects the dissolution/deposition process of Zn^2+^/Zn on the Zn anode surface. To investigate the effect of trace K^+^ on the Zn anode, three electrolyte systems with sufficient Zn^2+^ are selected for electrochemical tests: 1 m Zn^2+^ (gray), 0.9 m Zn^2+^ + 0.1 m K^+^ (red), and 1 m Zn^2+^ + 0.1 m K^+^ (blue). Notably, 0.1 m K^+^ is the optimal concentration matching the conventional working current density screened from the ZnHCF cathode side, which is fixed for anode verification after cathode gradient tests. As shown in Figure 6a–d, the Zn||Zn symmetric cell with 1 m Zn^2+^ + 0.1 m K^+^ electrolyte exhibits the optimal cycling stability at 0.4 mA·cm^−2^. The superior cycling stability of the 1 m Zn^2+^ + 0.1 m K^+^ system is ascribed mainly to two critical factors. The optimized solvation structure reduces the content of free water, thereby effectively inhibiting the parasitic hydrogen evolution side reactions [76]. Concurrently, the enhanced Zn^2+^ transport kinetics lowers the deposition polarization [77]. The CV profiles of Zn||Cu asymmetric cell (Figure 6e) and the EIS test of Zn||Zn symmetric cell (Figure 6f) further confirm this trend: the 1 m Zn^2+^ + 0.1 m K^+^ system (blue) exhibits the highest exchange current and the smallest polarization, along with the lowest resistance. Furthermore, the activity of the hydrogen evaluation reaction (HER) is revealed by the LSV test (Figure 6g): the order of the inflection point potentials of the curves shows that the 1 m Zn^2+^ + 0.1 m K^+^ system (blue) exhibits the best HER inhibitory effect, which is also verified by the order of the absolute values of the HER current density at the same negative potential. These results fully confirm the regulating effect of trace K^+^ on the Zn anode surface.

## 3 Conclusion

In summary, we propose a current‐dependent critical dissolution concentration mechanism to quantify the optimal K^+^ ratio in K‐Zn mixed electrolytes to achieve the best electrochemical performance of ZnHCF||Zn battery. Specifically, high concentration of K^+^ can induce spontaneous ZnHCF cathode dissolution by adjusting the reaction quotient *Q* in *Van't Hoff Isotherm equation*. Based on this, the current‐free ZnHCF soaking experiments confirm that a K^+^ concentration higher than 0.75 m triggers significant ZnHCF dissolution. Further, SEM and TEM confirm the same trend under current influence, and the ion enrichment effect leads to a similar ZnHCF dissolution reaction at a K^+^ concentration lower than the current‐free CDC (0.75K). XRD patterns and HRTEM images reveal the existence of an optimal K^+^ concentration at different current densities. Above this concentration, excessive K^+^ dissolves the ZnHCF lattice, leading to a decrease in layer distance. Below this concentration, insufficient K^+^ insertion fails to fully utilize the lattice support function. Additionally, long‐cycle data of ZnHCF||Zn batteries are fitted to obtain the optimal K^+^ concentration at any current density. Based on this curve, the specially selected K^+^ concentration (0.05K) at 350 mA·g^−1^ exhibits the optimal electrochemical performance at this current density, verifying the reliability of the curve for predicting the optimal K^+^ concentration. Besides, it is confirmed that trace amounts of K^+^ have a regulatory effect on the Zn anode. This work provides new insights for formulating aqueous K‐Zn mixed electrolytes, advancing theoretical understanding and engineering applications of ion competition.

## 4 Experimental Section

### 4.1 Cathode Preparation

Cathode active material Zn_3_[Fe(CN)_6_]_2_ (ZnHCF) was synthesized using a coprecipitation reaction. Specifically, 4 mmol of K_3_Fe(CN)_6_ and 4 mmol of ZnSO_4_·7H_2_O were dissolved in 400 mL of deionized water to obtain solutions A and B, respectively. After being completely dissolved, solutions A and B were simultaneously added dropwise to an empty beaker C. The mixed solution in beaker C was then stirred vigorously at room temperature for 24 h. After 24 h, stop stirring and let the suspension in beaker C stand at room temperature for 12 h to obtain a yellow‐brown solid. Then the obtained solids were filtered, washed with deionized water and anhydrous ethanol for three times, and then dried at 70° C until the powder turned green. The ZnHCF@C composite was fabricated via a one‐step water‐bath liquid‐phase reduction method using vitamin C as the reducing agent. Specifically, ZnHCF was dispersed in 8 mL of graphene oxide (GO) suspension via ultrasonic treatment for 30 min to form a homogeneous dispersion system. Then, 3 mL of aqueous solution containing 264 mg of L‐ascorbic acid (vitamin C, VC) was added under stirring, and the mixture was heated in a 95°C water bath for 2 h. The resulting precipitate was washed with deionized water several times and freeze–dried to finally obtain the ZnHCF@reduced graphene oxide (ZnHCF@RGO, denoted as ZnHCF@C) composite. Cathode electrodes were prepared by casting slurries of active materials (70 wt.%), acetylene black (20 wt.%), and polyvinylidene fluoride (10 wt.%) in n‐methyl‐2‐pyrrolidinone on Ti foil, and air drying at 60 C for 12h. Discs with a diameter of 1.3 cm were cut for electrochemical tests. The mass loading of the ZnHCF electrode is 1∼2 mg cm^−2^.

### 4.2 Anode Preparation

Commercial Zn foil (Qingyuan Metal Material) with a thickness of 0.08 mm is polished and washed with deionized water and 0.1 m HCl solution. Discs with a diameter of 1.3 cm were cut for electrochemical tests.

### 4.3 K‐Zn Mixed Electrolyte Preparation

First, prepare 0.5 m K_2_SO_4_ solution D and 1 m ZnSO_4_ solution E separately to ensure that the solutions contain 1 m K^+^ and 1 m Zn^2+,^ respectively. To obtain a K‐Zn mixed solution with a total cation concentration of 1 m, solutions D and E were mixed in the following molar ratios: K^+^:Zn^2+^ = 1:0, 0.9:0.1, 0.8:0.2, 0.78:0.22, 0.77:0.23, 0.75:0.25, 0.3:0.7, 0.2:0.8, 0.1:0.9, 0.05:0.95, 0.03:0.97, and 0:1, marked as 1K, 0.9K, 0.8K, 0.78K, 0.77K, 0.75K, 0.3K, 0.2K, 0.1K, 0.05K, 0.03K, 0K (also 1 Zn), respectively. In addition, a K‐Zn mixed solution of K^+^:Zn^2+^ = 0.1:1 was prepared to verify the effect of K^+^ in inhibiting the hydrogen evolution reaction.

### 4.4 Material Characterization

The crystalline structure was characterized by x‐ray diffraction (XRD, Bruker D8 Advance diffractometer with Cu Kα irradiation (λ = 1.54Å). Scanning electron microscopy (SEM, Crossbeam 350) and field‐emission TEM (FEI Tecnai G2 F30) were utilized to characterize the morphology of the cathode. Energy dispersive x‐ray spectrometer (EDS) images were collected by an Oxford Inca X‐Max EDX spectrometer. The elemental contents of Zn and Fe in the electrolyte were determined by an inductively coupled plasma optical emission spectrometer (ICP‐OES, Perkin Elmer Optima 2100 DV).

### 4.5 Electrochemical Measurements

ZnHCF||Zn full cells, Zn||Zn symmetric cells, and Zn||Cu asymmetric cells (CR2032‐type) were assembled in air using the Whatman glass microfiber separator at 25°C and stood for 12 h for electrochemical tests. The electrolytes employed herein were a series of K‐Zn mixed electrolytes. The galvanostatic charging–discharge (GCD) and long cycling performances of ZnHCF||Zn full cells at 100, 250, and 500 mA·g^−1^ were carried out by the LAND test systems (CT3001A). The cyclic voltammetry (CV) tests of ZnHCF||Zn full cells were conducted on an electrochemical workstation (CHI660E) with a scan rate of 2 mV·s^−1^. For Zn||Zn symmetric cells, the LAND test system was employed to evaluate the cycle performance at a current density of 0.4 mA·cm^−2^ and an areal capacity of 0.2 mAh·cm^−2^. The voltage‐time profiles were recorded for the cells at this current density. Linear scanning voltammetry (LSV) tests were conducted on the electrochemical workstation (CHI660E) with a scan rate of 1 mV·s^−1^. The same electrochemical workstation (CHI660E) was used to record electrochemical impedance spectra (EIS), in which the amplitude was 5 mV, and the frequency range was 100 kHz–10 mHz. For Zn||Cu asymmetric cells, the cyclic voltammetry (CV) tests were conducted on an electrochemical workstation (CHI660E) with a scan rate of 1 mV·s^−1^.

### 4.6 Homemade In Situ Optical Microscopies

The in situ ZnHCF||Zn optical microscopy observation device consists of in situ tank and ZnHCF (left) and Zn (right) square sheets cut into 1cm × 1 cm. Adding K‐Zn mixed electrolytes dropwise between two square sheets forms an open ZnHCF||Zn full cell. By connecting the two electrode sheets to an electrochemical workstation and applying a current density of 500 mA·g^−1^, the device can be continuously charged and discharged at a constant current. An optical micro‐camera (Belona, 200X–800X) was placed above the in situ tank to observe and record in real‐time the color changes in electrolyte during charge–discharge in the in situ device. The assembly and testing methods of the in situ ZnHCF||ZnHCF device are the same, except that one side of the Zn sheet is replaced with a ZnHCF sheet.

## Author Contributions

**Shuang Zheng**: investigation. **Yuqian Li**: conceptualization, methodology, formal analysis, funding acquisition, writing – original draft, writing – review and editing. **Wenju Wang**: validation, writing – review and editing, funding acquisition, project administration. **Yewei Li**: conceptualization, methodology, visualization, writing – original draft, data curation, software. **Jiyuan You**: visualization. **Yuchen Zhang**: software. **Yan Liu**: project administration, resources, writing – review and editing. **Yanhao Pan**: validation, supervision.

## Conflicts of Interest

The authors declare no conflicts of interest.

## Supporting information




**Supporting File**: advs75876‐sup‐0001‐SuppMat.docx.

## Data Availability

Data supporting the findings of this study are available from the corresponding author upon reasonable request.
