# Supplementary material for: Tailoring K+ Dosage in K+/Zn2+ Mixed Electrolytes via Lattice Expansion Regulation of Zinc Hexacyanoferrate for High‐performance Zinc Ion Batteries
Source: Adv Sci (Weinh). 2026 Jun 24:e75876. Online ahead of print. doi: 10.1002/advs.75876 (PMC13335831; doi:10.1002/advs.75876)
Supplement: Supplementary file 1 — Supporting File: advs75876‐sup‐0001‐SuppMat.docx. [file ADVS-9999-e75876-s001.docx]

Supporting Information

**Tailoring K^+^ dosage in K^+^/Zn^2+^ mixed electrolytes via lattice expansion regulation of zinc hexacyanoferrate for high-performance zinc ion batteries**

Yewei Li^a, b^, Yuqian Li^a,^ *, Yuchen Zhang^a^, Shuang Zheng^a^, Yanhao Pan^a^, Jiyuan You^c^, Yan Liu^b,^ *, Wenju Wang^a,^ *

^a^ *School of Energy and Power Engineering, Nanjing University of Science and Technology, Nanjing, 210094, China*.

^b^ *School of Chemistry and Chemical Engineering, Nanjing University of Science and Technology, Nanjing, 210094, China*.

^c^ *Huaneng Nanjing Jinling Power Generation Co.,Ltd. Nanjing, 210034, China*.

* Corresponding authors

*E-mail addresses:* [liyuqian@njust.edu.cn](mailto:liyuqian@njust.edu.cn) *(Y. Li),* [liuyan@njust.edu.cn](mailto:liuyan@njust.edu.cn) *(Y. Liu),*

[wangwenju@njust.edu.cn](mailto:wangwenju@njust.edu.cn) *(W. Wang).*

**Keywords**: PBA cathode, cation electrolyte additive, ion competition, aqueous zinc ion batteries, aqueous Zn based electrolyte

**
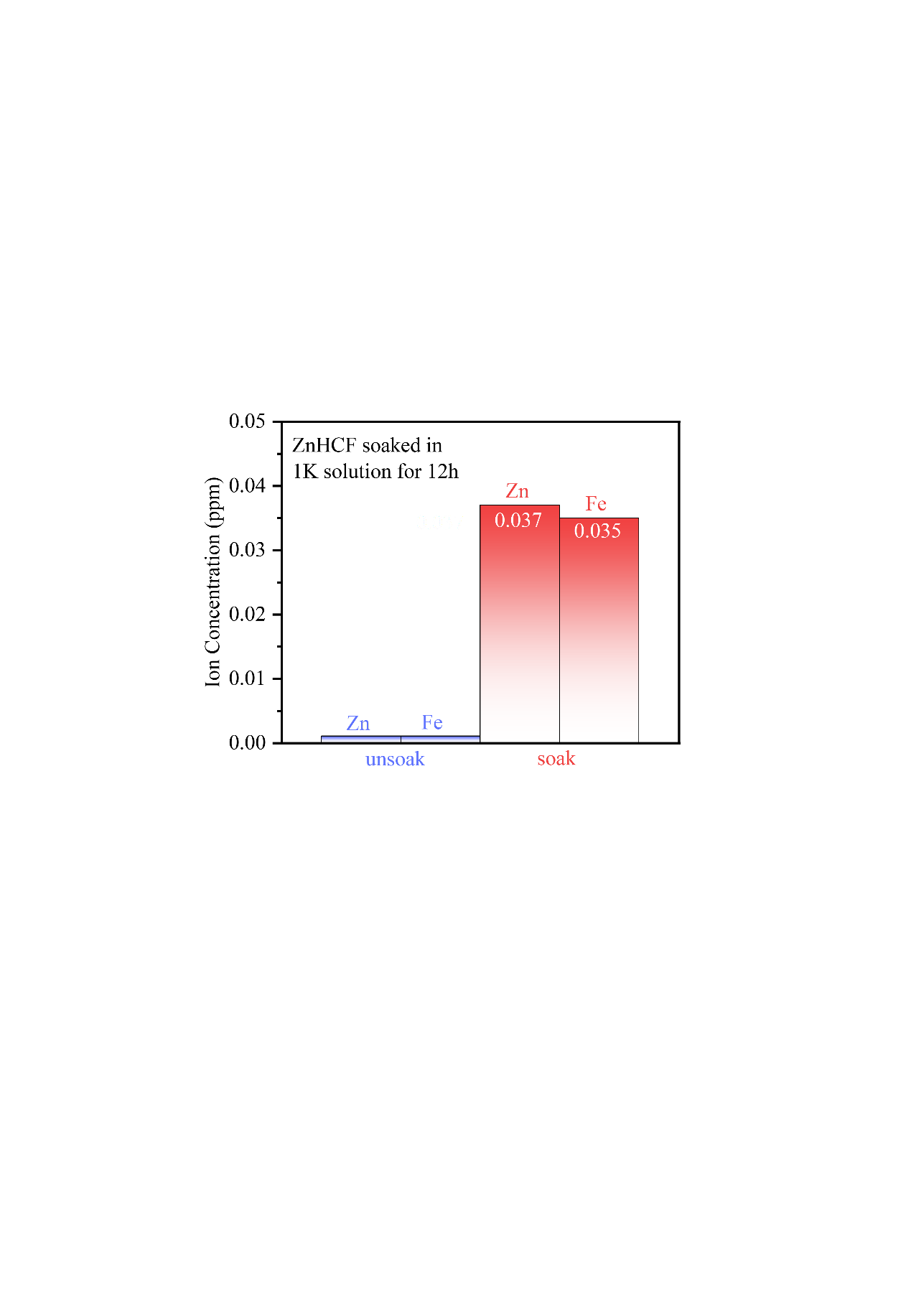
**

**Figure S1**. Zn and Fe elements contents in 1K solution after 12h soaking.


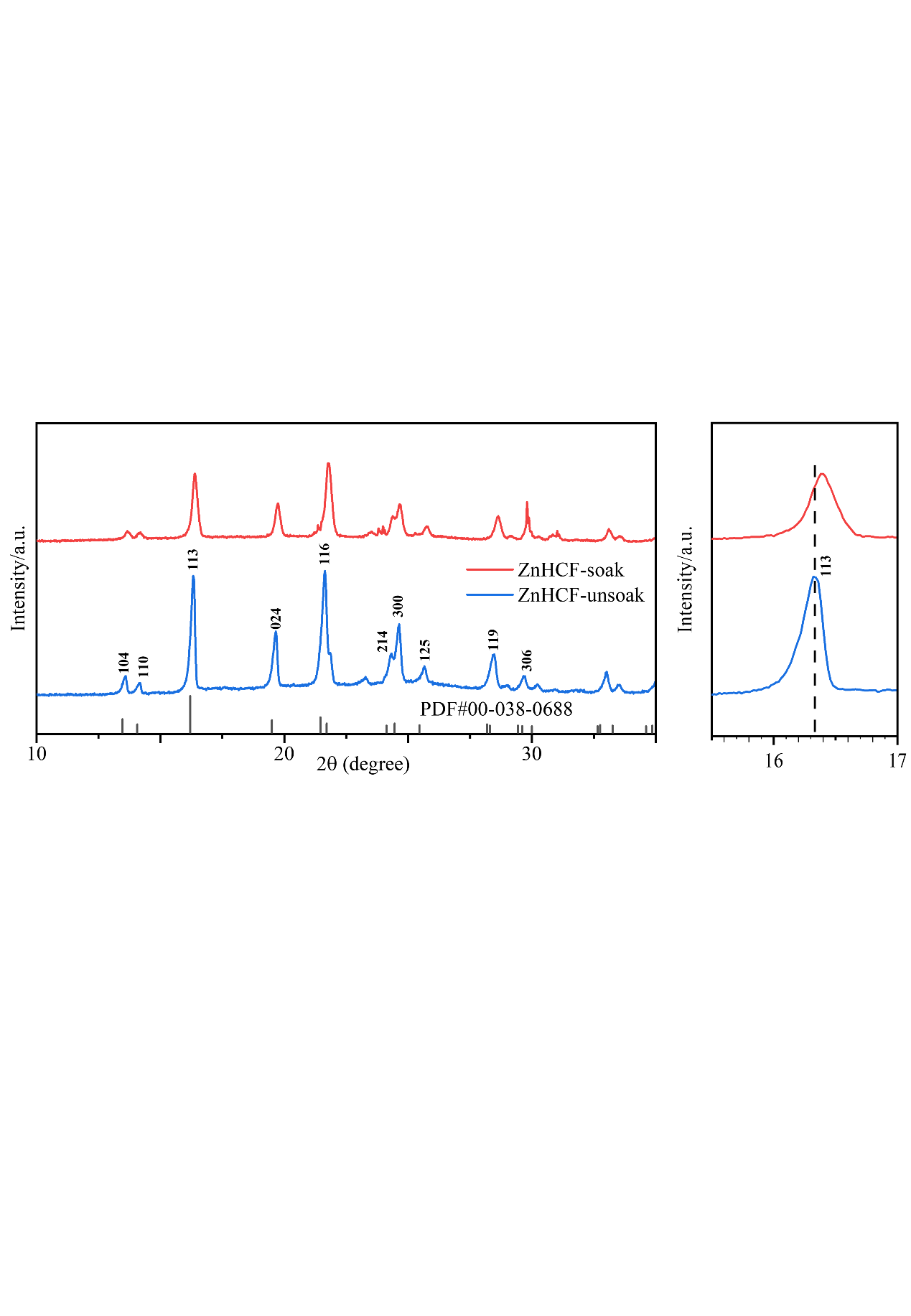


**Figure S2**. XRD patterns of ZnHCF soaked in 1K solution for 12h.


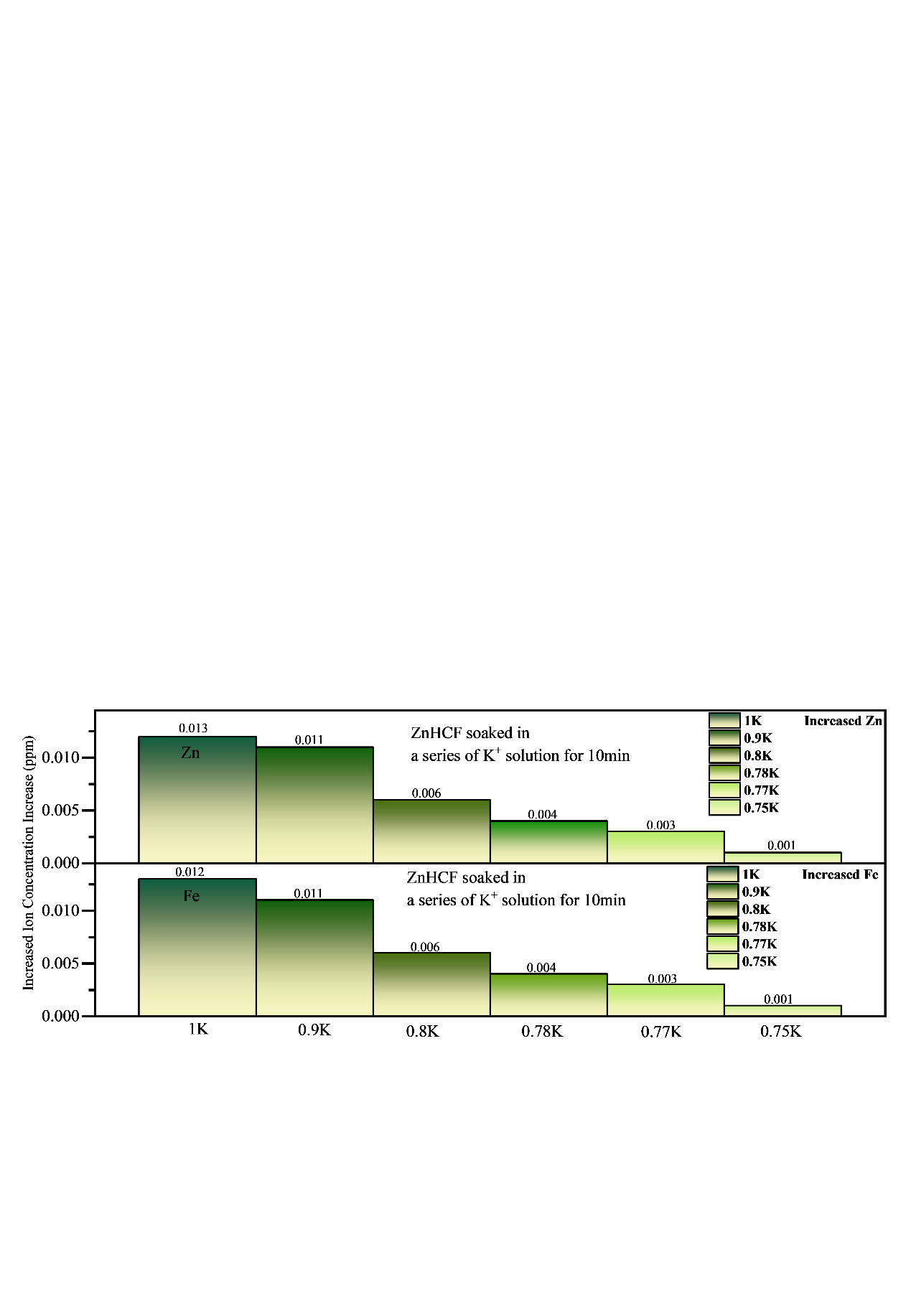


**Figure S3**. Zn and Fe elements contents in series of K-Zn mixed solution after 10 min soaking.


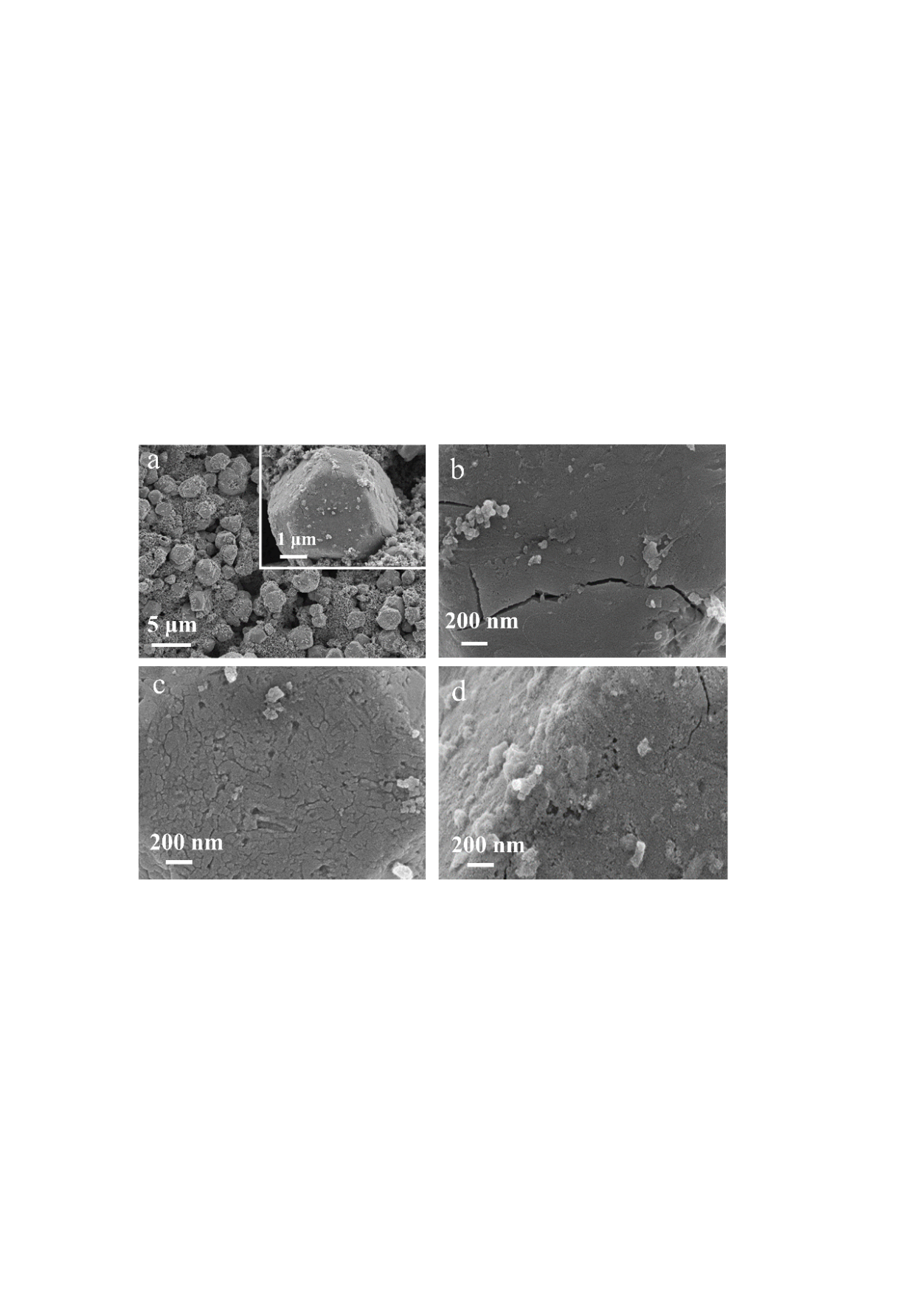


**Figure S4**. a) SEM images of cycled ZnHCF cathode using 0.05K electrolyte. Microcracks on ZnHCF surface using b) 0.2K, c) 0.1K and d) 0.05K electrolyte.


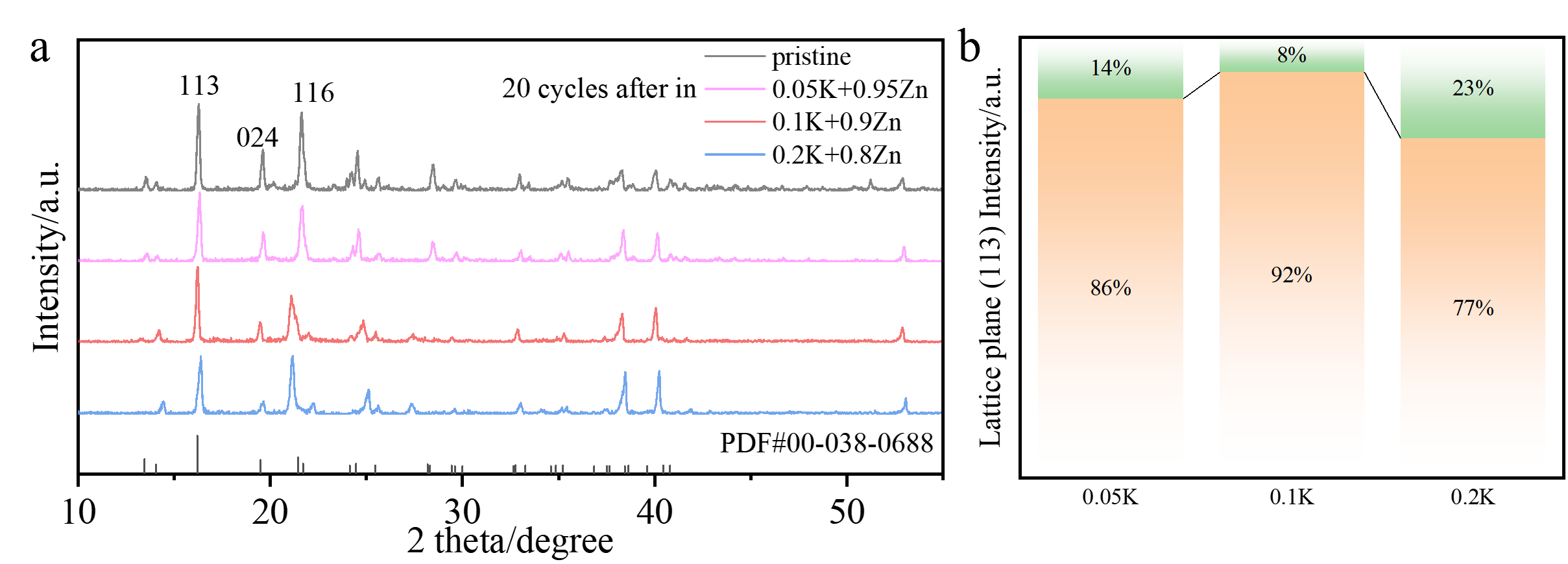


**Figure S5**. a) XRD patterns of pristine ZnHCF and cycled ZnHCF in 0.2K, 0.1K and 0.05K electrolyte. b) The retention degree (orange) and attenuation degree (green) of the peak intensity of the (113) crystal plane of the cycled ZnHCF cathode.


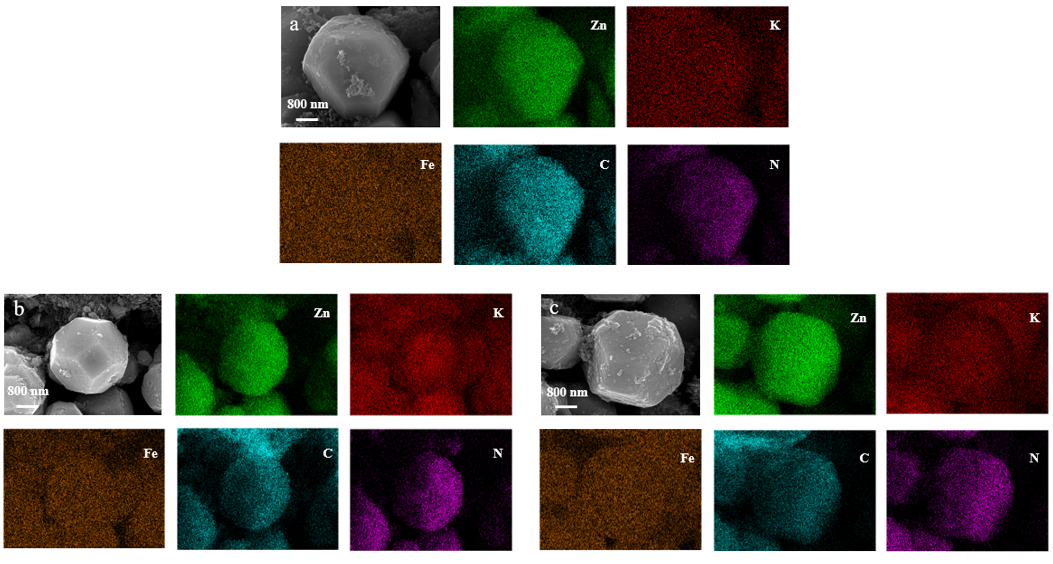


**Figure S6**. Elements distribution of cycled ZnHCF cathode using a) 0.2K electrolyte, b) 0.1K electrolyte and c) 0.05K electrolyte.


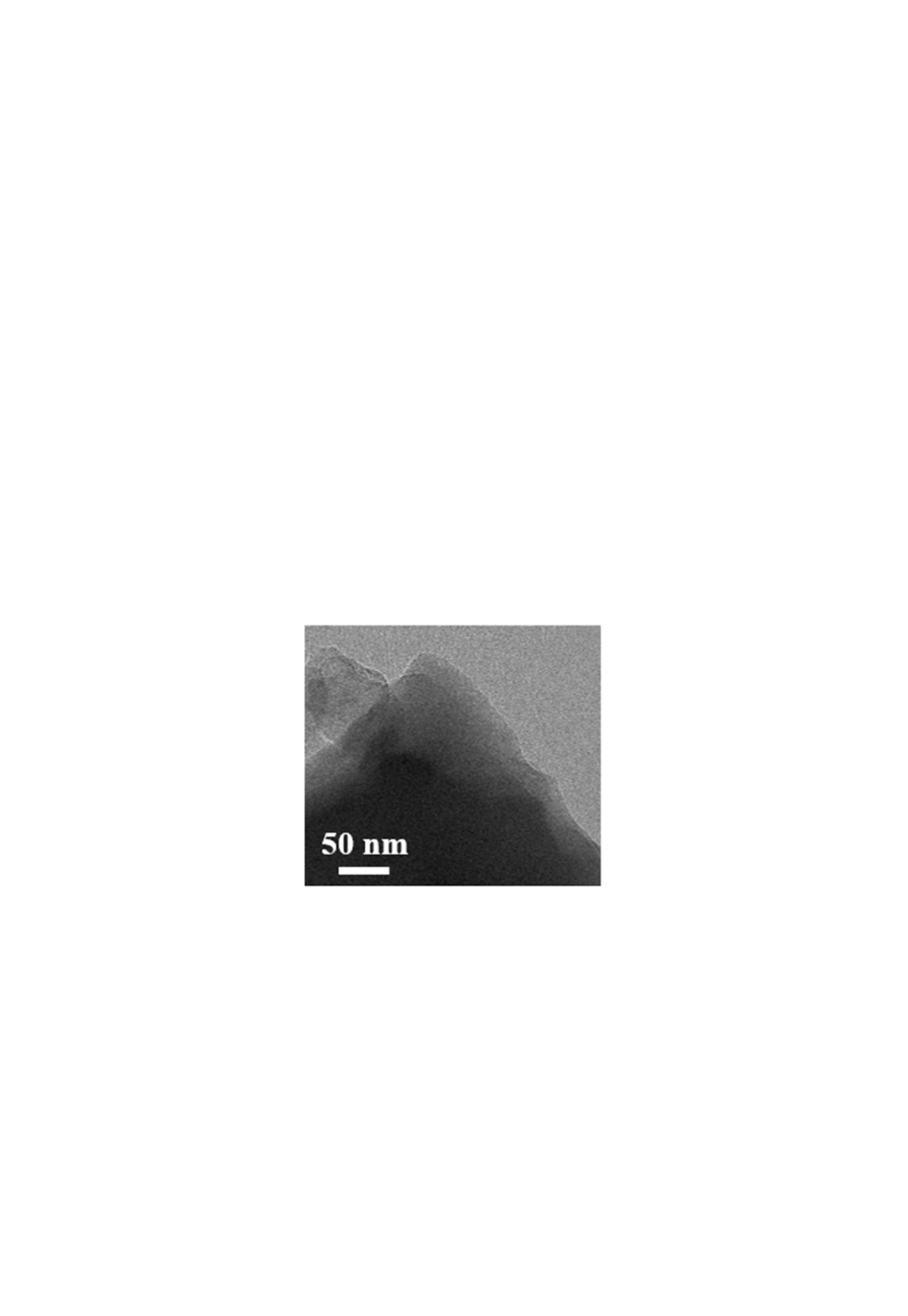


**Figure S7**. TEM images of cycled ZnHCF cathode using 0.2K electrolyte.


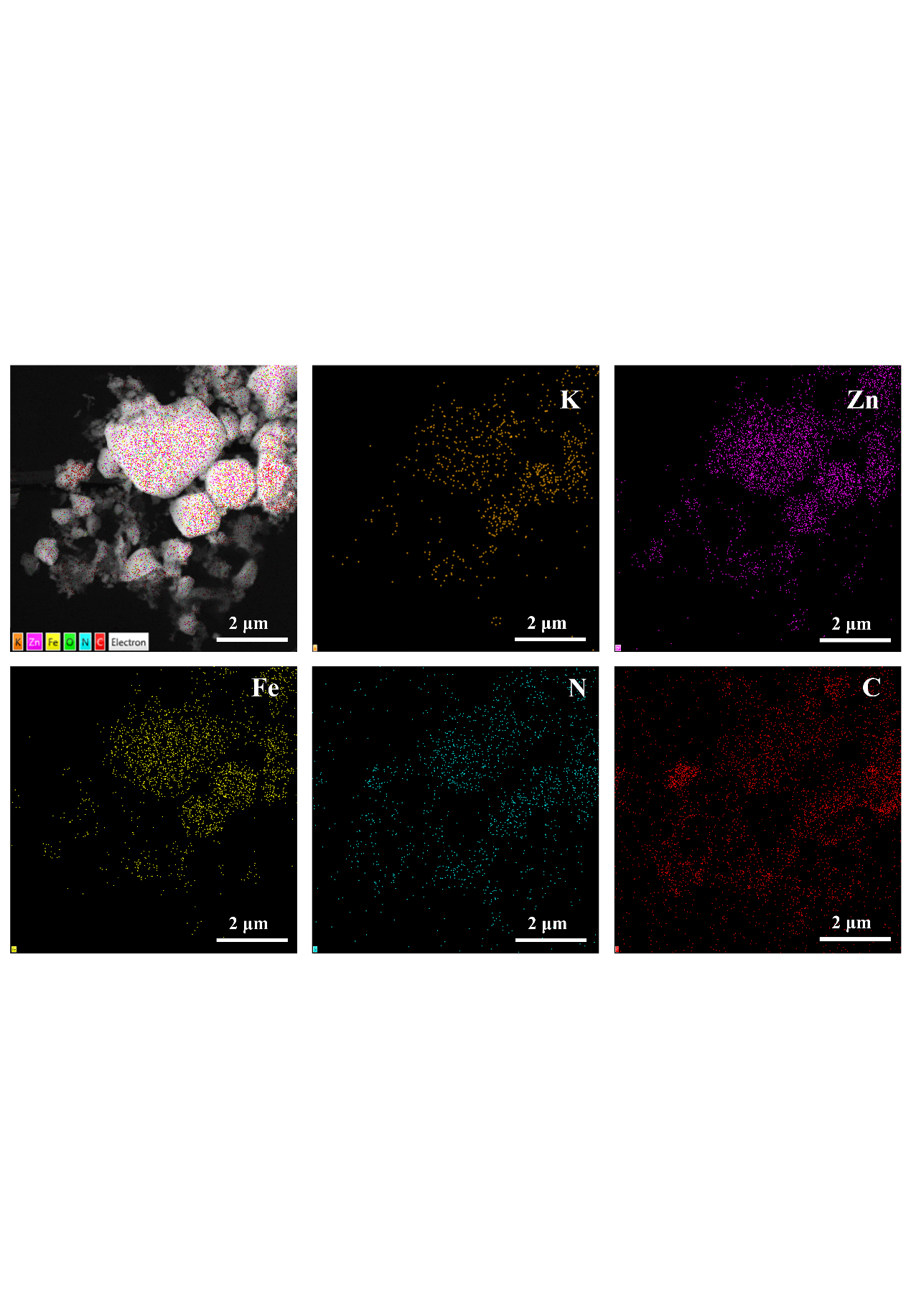


**Figure S8**. TEM-EDS images of cycled ZnHCF cathode using 0.1K electrolyte.


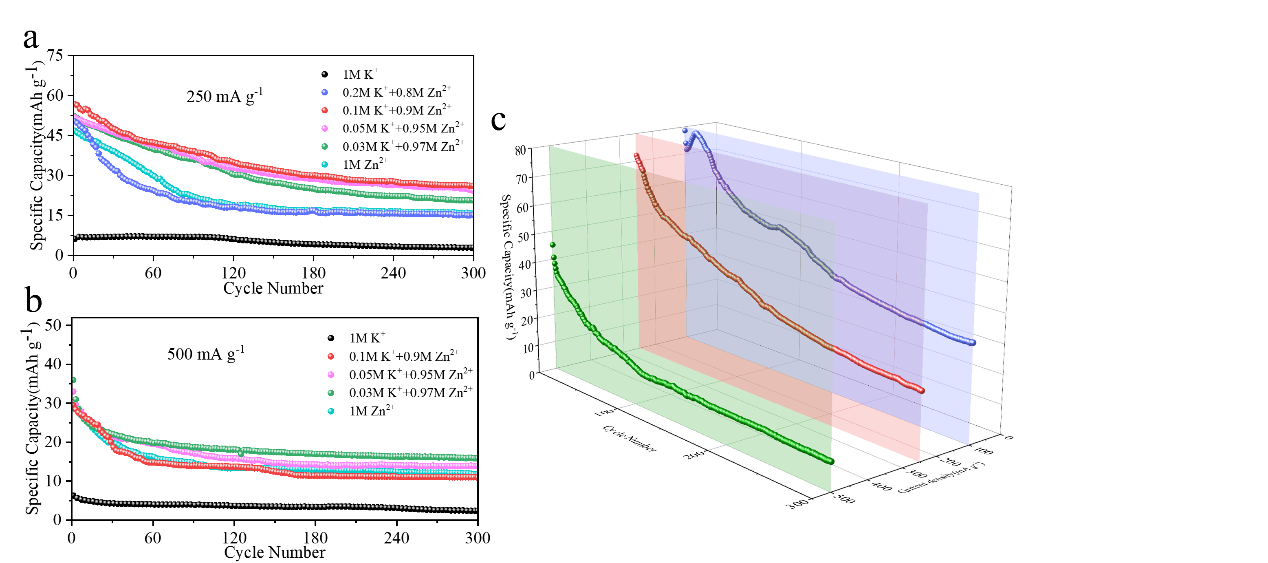


**Figure S9.** Long cycle performance at a) 250 mA·g^-1^, b) 500 mA·g^-1^, c) The critical K/Zn concentration corresponding to highest overall capacity at 100 mA·g⁻¹ (blue, 0.2K), 250 mA·g⁻¹ (red, 0.1K) and 500 mA·g⁻¹ (green, 0.03K).


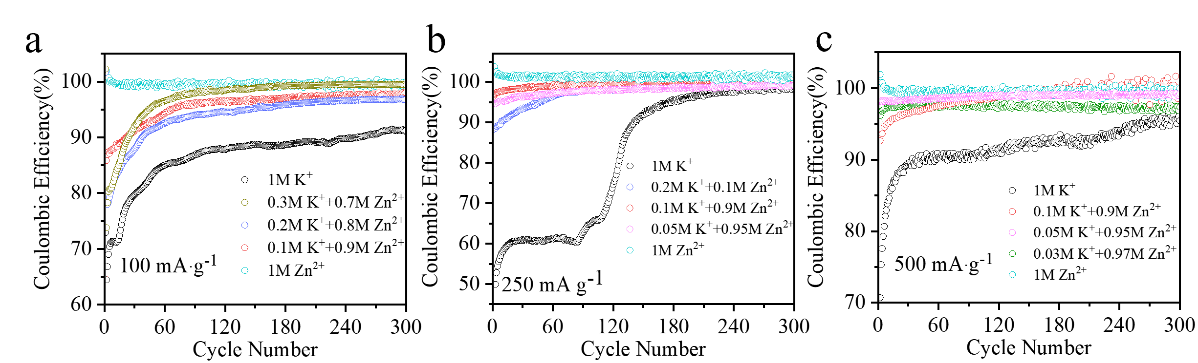


**Figure S10**. The coulombic efficiency at a) 100 mA·g^-1^, b) 250 mA·g^-1^ and c) 500 mA·g^-1^.


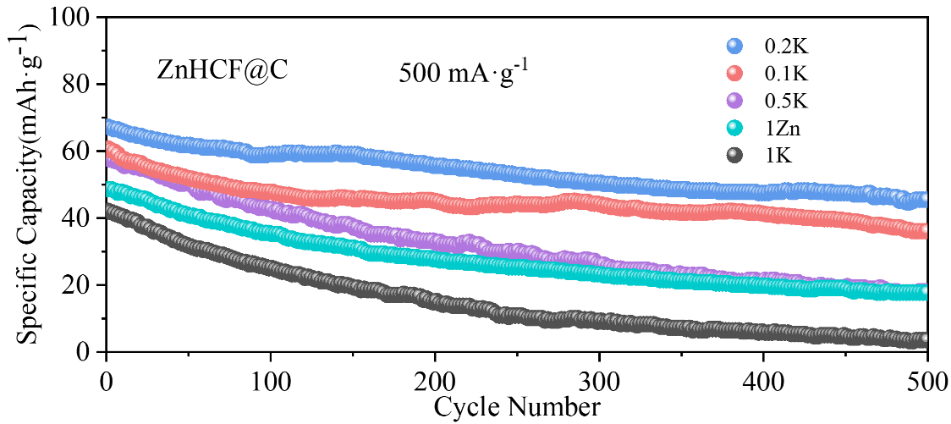


**Figure S11.** Long cycle performance of ZnHCF@C||Zn full battery at 500 mA·g^-1^.


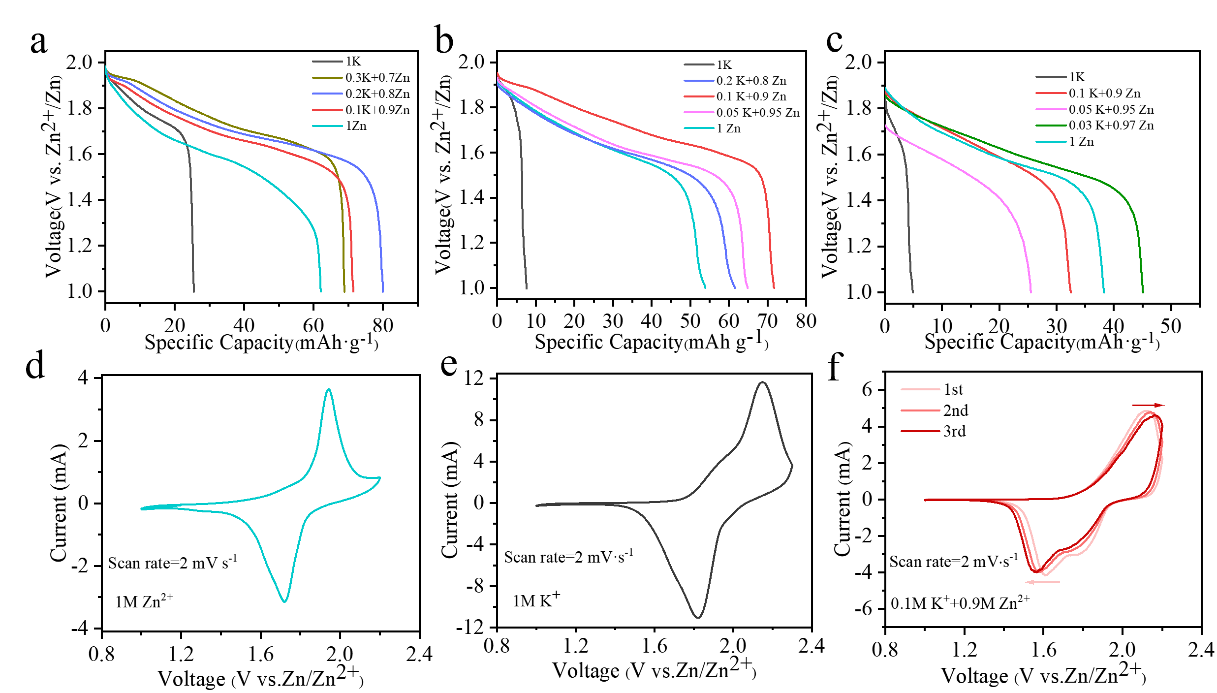


**Figure S12**. Galvanostatic charge-discharge curves at d) 100 mA·g^-1^, e) 250 mA·g^-1^ and f) 500 mA·g^-1^. CV profiles of the ZnHCF||Zn battery with a scan rate of 2 mV·s^-1^ in g) 1Zn, h) 1K and i) 0.1K electrolytes.


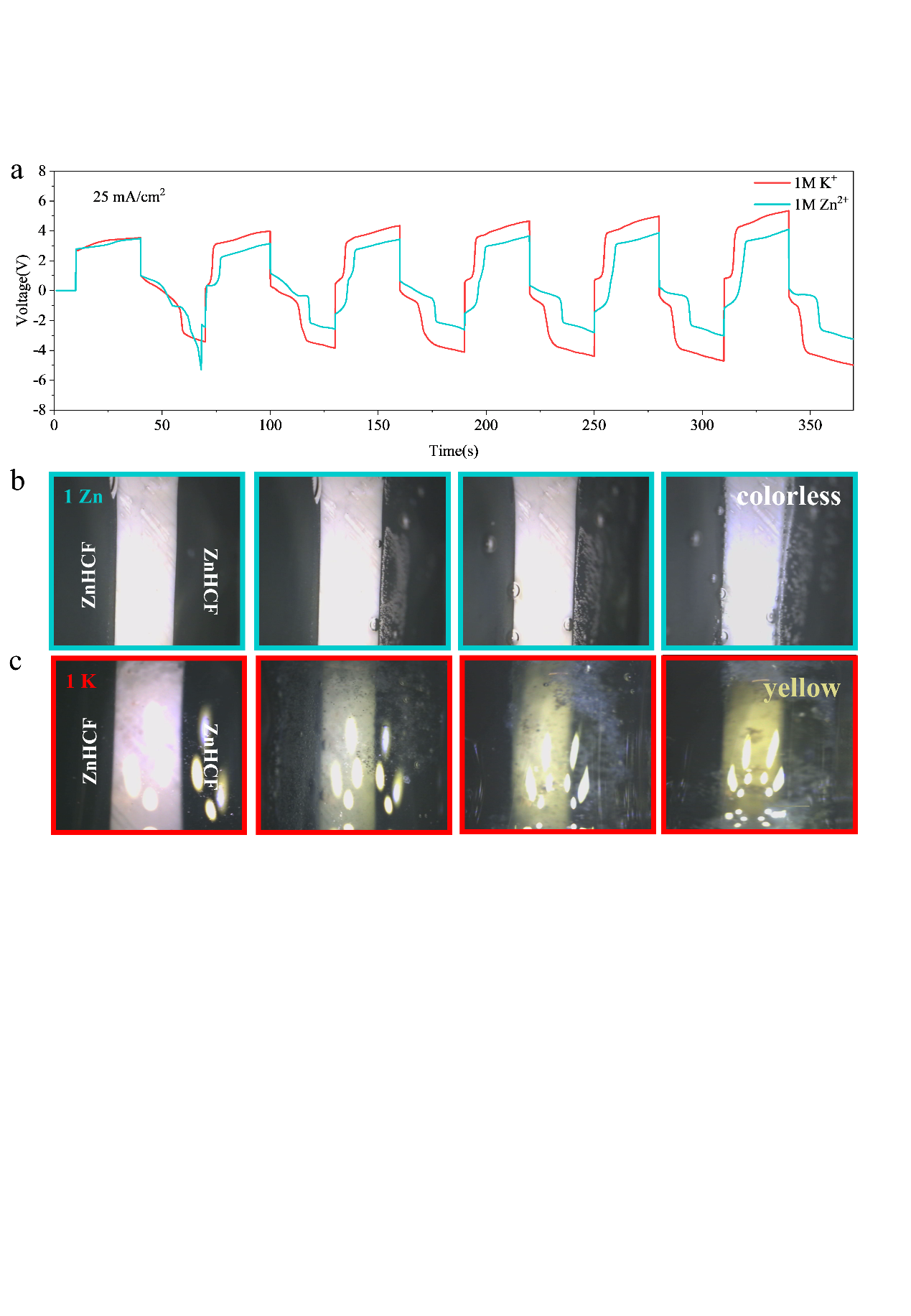


**Figure S13**. a) Galvanostatic charge-discharge curves of in-situ Zn||Zn symmetric cell using 1Zn and 1K electrolyte at 25 mA·cm^-2^. The color change of b) 1Zn and c) 1K electrolyte at 25 mA·cm^-2^.


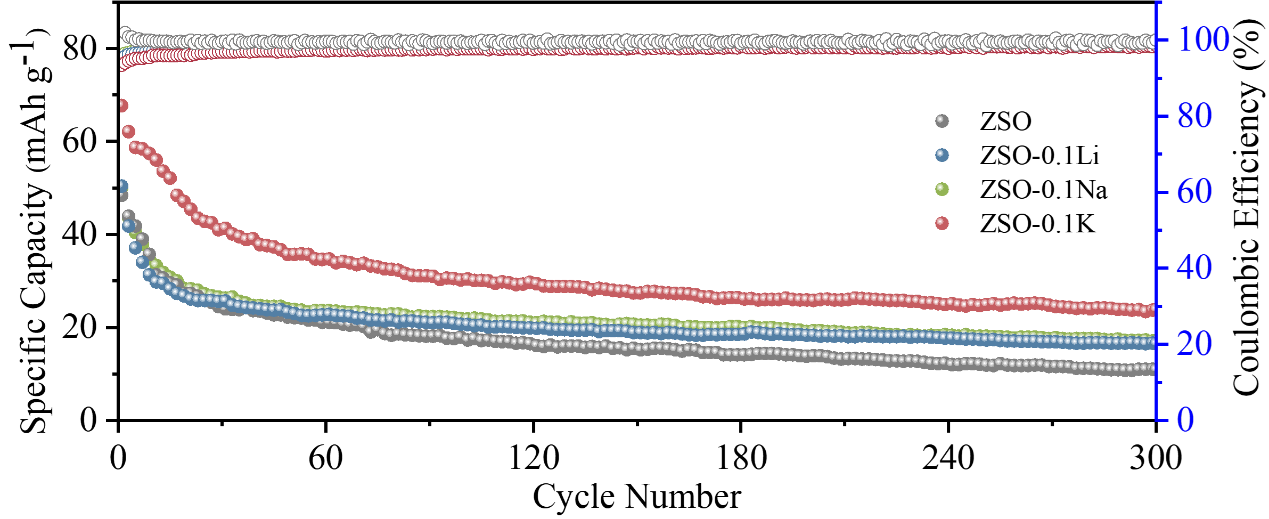


**Figure S14.** Long cycle performance of ZnHCF||Zn full batteries using different mixed electrolytes at 250 mA·g^-1^ (ZSO represents 1M ZnSO4, ZSO-0.1Li represents 0.9M ZnSO_4_+0.05M Li_2_SO_4_, ZSO-0.1Na represents 0.9M ZnSO_4_+0.05M Na_2_SO_4_, ZSO-0.1K represents 0.9M ZnSO_4_+0.05M K_2_SO_4_).

**Table S1**. Elements contents of cycled ZnHCF cathode using 0.2K, 0.1K and 0.05K electrolyte.

| Element | 0.2K  (atomic %) | 0.1K  (atomic %) | 0.05K  (atomic %) |
| --- | --- | --- | --- |
| K | 2.09 | 3.04 | 0.80 |
| Zn | 4.79 | 4.84 | 5.74 |
| Fe | 3.89 | 4.21 | 4.57 |
| C | 57.65 | 57.75 | 56.78 |
| N | 31.58 | 30.16 | 32.11 |

**Table S2**. ICP-OES results of cycled ZnHCF cathode using 0.2K, 0.1K and 0.05K electrolyte.

| Sample | K  (mg·L^-1^) | Zn  (mg·L^-1^) | Fe  (mg·L^-1^) | Mole ratio of  K: Zn: Fe |
| --- | --- | --- | --- | --- |
| 0.2K | 6.11 | 24.47 | 14.74 | 1.18: 2.82: 2 |
| 0.1K | 8.99 | 29.89 | 17.50 | 1.47: 2.93: 2 |
| 0.05K | 2.88 | 30.67 | 18.04 | 0.46: 2.91: 2 |
